# Guanylyl Cyclase A/cGMP Signaling Slows Hidden, Age- and Acoustic Trauma-Induced Hearing Loss

**DOI:** 10.3389/fnagi.2020.00083

**Published:** 2020-04-09

**Authors:** Philine Marchetta, Dorit Möhrle, Philipp Eckert, Katrin Reimann, Steffen Wolter, Arianna Tolone, Isabelle Lang, Markus Wolters, Robert Feil, Jutta Engel, François Paquet-Durand, Michaela Kuhn, Marlies Knipper, Lukas Rüttiger

**Affiliations:** ^1^Molecular Physiology of Hearing, Tübingen Hearing Research Centre, Department of Otolaryngology, University of Tübingen, Tübingen, Germany; ^2^Department of Anatomy and Cell Biology, Schulich School of Medicine and Dentistry, University of Western Ontario, London, ON, Canada; ^3^Cell Death Mechanisms Group, Institute for Ophthalmic Research, Centre for Ophthalmology, University of Tübingen, Tübingen, Germany; ^4^Department of Biophysics, Center for Integrative Physiology and Molecular Medicine, Hearing Research, Saarland University, Homburg, Germany; ^5^Signal Transduction and Transgenic Models, Interfaculty Institute of Biochemistry, University of Tübingen, Tübingen, Germany; ^6^Institute of Physiology, University of Würzburg, Würzburg, Germany

**Keywords:** inner ear, cGMP, otoprotection, guanylyl cyclase A, aging, hidden hearing loss, PARP-1, KCNQ4

## Abstract

In the inner ear, cyclic guanosine monophosphate (cGMP) signaling has been described as facilitating otoprotection, which was previously observed through elevated cGMP levels achieved by phosphodiesterase 5 inhibition. However, to date, the upstream guanylyl cyclase (GC) subtype eliciting cGMP production is unknown. Here, we show that mice with a genetic disruption of the gene encoding the cGMP generator GC-A, the receptor for atrial and B-type natriuretic peptides, display a greater vulnerability of hair cells to hidden hearing loss and noise- and age-dependent hearing loss. This vulnerability was associated with GC-A expression in spiral ganglia and outer hair cells (OHCs) but not in inner hair cells (IHCs). GC-A knockout mice exhibited elevated hearing thresholds, most pronounced for the detection of high-frequency tones. Deficits in OHC input–output functions in high-frequency regions were already present in young GC-A-deficient mice, with no signs of an accelerated progression of age-related hearing loss or higher vulnerability to acoustic trauma. OHCs in these frequency regions in young GC-A knockout mice exhibited diminished levels of KCNQ4 expression, which is the dominant K^+^ channel in OHCs, and decreased activation of poly (ADP-ribose) polymerase-1, an enzyme involved in DNA repair. Further, GC-A knockout mice had IHC synapse impairments and reduced amplitudes of auditory brainstem responses that progressed with age and with acoustic trauma, in contrast to OHCs, when compared to GC-A wild-type littermates. We conclude that GC-A/cGMP-dependent signaling pathways have otoprotective functions and GC-A gene disruption differentially contributes to hair-cell damage in a healthy, aged, or injured system. Thus, augmentation of natriuretic peptide GC-A signaling likely has potential to overcome hidden and noise-induced hearing loss, as well as presbycusis.

## Introduction

Hearing loss is considered the fourth leading cause of disability worldwide and is one of the most common conditions affecting older people. Peripheral age-dependent hearing loss has recently been defined as a severe but amendable risk factor for the development of dementia. Thus, treatment of hearing deficits can be important for cognitive health^1^ (Livingston and Frankish, 2015; Fitzakerley and Trachte, 2018).

As we age, hearing sensitivity gradually and progressively declines. The presbycusis refers to a progressive, age-dependent hearing loss that results from loss of outer hair cell (OHC) function. This decline in OHC function typically begins in regions that respond to high-frequency sounds (Frisina, 2009; Frisina and Frisina, 2013; Lee, 2015). The majority of the aging population experiences difficulties in perceiving speech in noise, even if audiometric thresholds are still normal or at least appear to be within the normal range, a phenomenon called hidden hearing loss (Füllgrabe et al., 2014). As demonstrated in rodents (Kujawa and Liberman, 2009; Rüttiger et al., 2013; Sergeyenko et al., 2013; Möhrle et al., 2019) and humans (Viana et al., 2015; Wu et al., 2019), hidden hearing loss is linked to a synaptopathy of the inner hair cell (IHC) synapse, the first synapse in the auditory system between the sensory cell and the afferent axon of the spiral ganglion neuron (SGN). IHC synaptopathy and auditory neuropathy precede presbycusis and progress with age (Sergeyenko et al., 2013; Möhrle et al., 2016). The protection of IHC and OHC function and therapeutic counteraction of noise-induced or age-dependent hearing loss may therefore be vital for maintaining speech comprehension and for the preservation of central auditory, or even cognitive, functions (Livingston et al., 2017). Accordingly, there is an urgent need for new pharmacological prevention strategies that have the potential to preserve cochlear hair cells and auditory fibers during aging and in response to the daily noise burden.

There is evidence that the genetics and function of cochlear cyclic guanosine monophosphate (cGMP)-forming guanylyl cyclases (GCs) play a fundamental role in normal hearing and cochlear pathophysiology (Fitzakerley and Trachte, 2018). A protective role of the cGMP-dependent protein kinase I (cGKI) signaling cascade for IHC synapses and OHCs was shown in rodent models of noise-induced damage (Jaumann et al., 2012). However, the upstream signaling pathways driving cGMP/cGKI signaling could not be sufficiently linked neither with the soluble GC (sGC) activated by nitric oxide (NO) (Möhrle et al., 2017), nor with the transmembrane, particulate GC-B, also named natriuretic peptide (NP) receptor NPR-B, activated by C-type NP (CNP) (Wolter et al., 2018). Using reverse transcription PCR analysis of cochlear tissue, the transmembrane, particulate GC-A, also named NPR-A, and its peptide ligand atrial NP (ANP) have been shown to be expressed in cochlear hair cells, supporting cells, and SGN (Krause et al., 1997; Suzuki et al., 1998; Dornhoffer et al., 2002; Qiao et al., 2011; Möhrle et al., 2017). The second specific GC-A ligand, B-type NP (BNP), was suggested to be absent from the inner ear (Suzuki et al., 1998; Shen et al., 2015).

GC-A is also expressed in various organs, such as the kidney, lung, adrenal gland, vasculature, brain, liver, endothelial and adipose tissues, or heart, and its fundamental role in cardiorenal biology is well known (reviewed in Potter, 2011). Thus, GC-A null mice exhibit cardiac hypertrophy, high blood pressure, and ventricular fibrosis (reviewed in Kuhn, 2016; Pandey, 2019). Also, GC-A activators have emerged as potential renal protective therapies, most importantly for the prevention and treatment of acute kidney injury (Chen and Burnett, 2018). The protective function of GC-A is based on the activation by the GC-A ligands ANP and BNP — both of these NPs bind to GC-A. These peptides have emerged as key regulators for energy consumption and metabolism, since they promote lipid oxidation and mitochondrial respiration (Kuhn, 2016).

Whether GC-A displays a protective role for hearing by stimulating the cGMP/cGKI signaling cascade remains elusive. Because ANP and BNP have emerged as key regulators of energy consumption and metabolism (Ramos et al., 2015), we suggest that GC-A signaling in the inner ear is important for metabolic supply as well. Several findings indicate that under conditions of acoustic trauma (AT), aging, metabolic demand, mitochondrial dysfunction, oxidative stress (Fujimoto and Yamasoba, 2019), or activation of hormonal stress responses (Singer et al., 2013, 2018; Fetoni et al., 2019; Möhrle et al., 2019; Prasad and Bondy, 2020) particularly the cochlear partitions corresponding to high-frequency sound processing are affected. If GC-A and activation by its ligands ANP and BNP shall have a protective role for IHC synapses and OHC function to prevent loss of hearing after loud sound exposure or aging, we hypothesize that GC-A gene disruption will negatively influence hearing function with stronger effects in the cochlear regions that are most sensitive to damage. We therefore focused our study on high-frequency representing cochlear partitions (mid-basal and basal cochlear turns) and analyzed the impact of a genetic GC-A (*Npr1*) disruption in GC-A knockout (KO) mice of different ages on hearing and hearing loss after noise exposure.

We included the analysis of the characteristic features of OHC and IHC phenotypes. Both types of sensory hair cells respond to cGMP upregulation through phosphodiesterase (PDE) 5 inhibitors (Jaumann et al., 2012). This encompasses the analysis of the membrane-bound potassium channel KCNQ4, a member of the voltage-gated channel subfamily (KQT member 4) that mediates the dominating K^+^ current in OHCs, *I*_K,n_ (Marcotti and Kros, 1999). Furthermore, we quantified poly (ADP-ribose) (PAR) polymers, products of PAR polymerase (PARP) activity and abundance (Paquet-Durand et al., 2007), and CtBP2/RIBEYE immunoreactivity at the basal IHC pole, where it labels the ribbon structures associated with synaptic vesicles (Kujawa and Liberman, 2009). Both KCNQ4 and PAR were shown to be linked with cGMP signaling in a previous study where PDE 5 inhibition by vardenafil led to increased PAR concentrations. The increased PAR concentrations were suggested to be responsible for persistent KCNQ4 staining after acoustic overexposure, due to DNA repair mechanisms (Jaumann et al., 2012). In that previous study, the vardenafil-induced elevation of PAR concentrations was accompanied by a healthy, “rescued” phenotype, as shown by persistent KCNQ4 staining in the OHCs and the maintenance of ribbons in the IHC synapses (Jaumann et al., 2012). To underscore a possible role of GC-A in the inner ear, we examined GC-A ligand ANP and BNP expression in specific cell types of the cochlea [IHC, OHC, spiral ganglia (SG)].

While we found hearing thresholds in young GC-A KO mice to be normal, a systematic longitudinal investigation of hearing function in young, middle-aged, and old GC-A KO mice with either sham exposure or AT revealed an earlier age-dependent hearing loss in comparison to wild-type (WT) littermates. The fine-structure analysis [auditory brainstem response (ABR) wave amplitude] of hearing function in GC-A KO mice, compared to GC-A WT mice, identified for the first time a differential contribution of GC-A to OHC and IHC damage in response to aging and AT. These findings may prompt future preclinical tests to assess the use of ANP/GC-A/cGMP signaling augmentation as an intervention strategy to counteract age- and noise-induced hearing loss (NIHL).

## Materials and Methods

### Generation of GC-A KO Mice

Mice with global gene disruption of GC-A (GC-A KO, 129-Npr1^tm1Gar/J^) were generated on a genetic background of C57BL/6 as previously described (Lopez et al., 1995). The mice were taken from the colony of Prof. Michaela Kuhn (Würzburg, Germany) and bred in the animal facility of the institute in Tübingen. Adult female and male GC-A KO mice and their WT littermates, as controls, were studied. Animals were bred by crossing heterozygous GC-A parental animals. They were housed in the animal care facility of the Department of Otolarynglogy, University of Tübingen (Germany), where noise levels did not exceed 50–60 dB sound pressure level (SPL)_rms_. Animals from three different age groups [2–4 months (young), 7–12 months (middle-age), and 16–18 months (old)] were studied. Mice were held in groups of one (only fighting males) to five mice in standard Macrolon polycarbonate cages containing nesting material, food (Altromin, 1324 BEST), and water *ad libitum* under a 12 h light–dark schedule (daylight period from 6 am to 6 pm). Animal care, procedures, and treatments were performed in accordance with institutional and national guidelines following approval by the University of Tübingen, Veterinary Care Unit, and the Animal Care and Ethics Committee of the regional board of the State Government of Baden-Württemberg, Germany, and followed the guidelines of the EU Directive 2010/63/EU for animal experiments (number: HN3/14).

### Hearing Measurements: Auditory Brainstem Response (ABR) and Distortion Product Otoacoustic Emission (DPOAE)

The auditory brainstem response (ABR) evoked by short-duration sound stimuli represents the summed activity of neurons in distinct anatomical structures along the ascending auditory pathway (Burkard and Don, 2007) and is measured by averaging the evoked electrical response recorded via subcutaneous cranial electrodes. ABR to click and noise-burst stimuli and the distortion product otoacoustic emission (DPOAE) for f2 = 1.24^∗^f1 kHz and L2 = L1-10 dB were recorded under anesthesia [0.05 mg/kg Fentanyl (Fentanyl-ratiopharm^®^ ratiopharm GmbH, Ulm, Germany), 0.5 mg/kg Medetomidin hydrochloride (Sedator, Eurovet Animal Health B.V., Bladel, Netherlands), 2.5 mg/kg Midazolam (Midazolam-hameln^®^; Hameln Pharma plus GmbH, Hameln, Germany), 0.2 mg/kg atropine (Atropinsulfat B.Braun, Melsungen, Germany)] in a soundproof chamber (IAC, Niederkrüchten, Germany), as previously described (Engel et al., 2006). In short, ABR thresholds were elicited with click (100 μs), noise-burst (1 ms duration), or pure-tone stimuli (3 ms, including 1 ms cosine squared rise and fall envelope, 2–45.2 kHz). OHC function was assessed by the DP-gram and growth function of the 2f1-f2 DPOAE (Knipper et al., 2000; Engel et al., 2006). Sound from two loudspeakers (Beyerdynamic DT-911, Heilbronn, Germany), and a probe microphone (Brüel & Kjaer 4135; preamplifier Brüel & Kjaer 2670, Naerum, Denmark) were directly channeled into the ear canal. Distortion product emission signals were recorded during a 260 ms sound presentation and averaged four times for each combination of sound pressure and frequency. The 2f1-f2 distortion product amplitude was measured for L1 ranging from 0 to 60 dB SPL at frequencies of f2 between 4 and 32 kHz. The frequencies f1 and f2 differ by a defined octave distance (f2/f1 = 1.24) and sound pressure (L1 = L2 + 10 dB). For the growth function at f2 = 5.6 or 11.3 kHz, only the OHC responses up to stimulus levels of 45 dB SPL were considered. Above 50 dB SPL stimulus level (L1), response compression by stereocilial non-linearity [saturation of mechanoelectrical transducer (MET) channels] and efferent feedback must be considered. We therefore limited the analysis to within the range up to 45 dB SPL, at which stimulus level the maximum contribution of OHC motility to the amplification of the basilar membrane movement is expected.

### Noise Exposure

Acoustic trauma was induced by exposing mice to broadband noise (8–16 kHz, 120 dB SPL_rms_ for 40 min) under anesthesia (see above), as previously described (Jaumann et al., 2012). The degree of the ABR threshold shift was measured approximately 30 min after sham or noise exposure to estimate temporary threshold shifts, and again after 7 days when noise-induced permanent threshold shifts had settled and further recovery from damage would no longer be expected (Liberman, 1980). Sham-exposed animals were anesthetized and placed in the chamber, but not exposed to the acoustic stimulus.

### Tissue Preparation

For cochlear cross-section immunohistochemistry, cochleae were isolated, fixed by immersion in 2% paraformaldehyde, 125 mM sucrose in 100 mM phosphate buffered saline, pH 7.4, for 2 h, and then decalcified for 45 min in RDO rapid decalcifier (Apex Engineering Products Corporation, Aurora, IL, United States) as previously described (Knipper et al., 1999; Zuccotti et al., 2012; Duncker et al., 2013; Singer et al., 2013), cryosectioned at 10 μm, and mounted on SuperFrost^∗^/plus microscope slides before storage at –20°C. For whole-mount immunohistochemistry, temporal bones of mature mice were dissected on ice and fixed using Zamboni’s fixative as described (Duncker et al., 2013).

### Immunohistochemistry

For immunohistochemistry, mouse cochlear sections were stained as previously described (Tan et al., 2007; Zuccotti et al., 2012; Duncker et al., 2013; Singer et al., 2013). Antibodies against prestin (rabbit, diluted 1:3000, for antibodies see Table 3) (Weber et al., 2002), potassium voltage-gated channel subfamily KQT member 4 (mouse, diluted 1:50) (StressMarq, Victoria, BC, Canada; Kharkovets et al., 2006), and C-terminal-binding protein 2 (CtBP2)/RIBEYE (rabbit, diluted 1:1500; ARP American Research Products, Inc.^TM^, Waltham, MA, United States; Uthaiah and Hudspeth, 2010) were used. Primary antibodies were detected using appropriate Cy3- (1:1500, Jackson Immuno Research Laboratories, West Grove PA, United States) or Alexa488-conjugated secondary antibodies (1:500, Invitrogen Molecular Probes, Paisley, United Kingdom). For double-labeling studies, both antibodies were simultaneously incubated for identical time periods. Sections and whole-mount preparations were viewed as previously described (Zampini et al., 2010) using an Olympus BX61 microscope (Olympus, Hamburg, Germany) equipped with epifluorescence illumination and analyzed with CellSens Dimension software (OSIS GmbH, Münster, Germany). To increase spatial resolution, slices were imaged over a distance of 15 μm within an image-stack along the *z*-axis (*z*-stack), followed by three-dimensional deconvolution using cellSens Dimension’s built-in algorithm.

### Colocalization of mRNA and Protein in Cochlea Whole-Mounts

mRNA (GC-A) and protein (Tuj-1) were colocalized on cochlear whole-mounts, as previously described (Singer et al., 2014). In brief, following prehybridization for 1 h at 37°C, sections were incubated overnight with GC-A riboprobes (for: 5′-TGT GAA ACG TGT GAA CCG GA-3′ and rev: 5′-AGG CGG ATC GTT GAA AGG G-3′) at 56°C, incubated with anti-digoxigenin antibody conjugated to alkaline phosphatase (anti-Dig-AP, Roche, Germany, 11093274910), and developed as previously described (Singer et al., 2013). For protein detection, streptavidin–biotin was blocked according to the manufacturer’s instructions (Streptavidin–Biotin Blocking Kit, Vector Laboratories, United States). Sections were incubated overnight at 4°C with the primary antibodies against Tuj-1 (1:500; monoclonal mouse Biozol MMS-435P), followed by incubation with the secondary antibody (1:500; biotinylated goat anti-rabbit, Vector Laboratories, BA-1000), streptavidin–horseradish peroxidase (1:300 in 1% BSA; Vector Laboratories, Burlingame, CA, United States), and chromogenic detection (AEC, 3-amino-9-ethylcarbazole, Vector Laboratories, SK-4200). Sections were cover slipped with gelatin, and analyzed using a BX61 microscope (Olympus, Hamburg, Germany).

### ABC/DAB Immunostaining in Cochlear Sections

The DAB staining was performed as described (Paquet-Durand et al., 2007). In short, the cochlear sections were put in a quenching solution containing H_2_O_2_, Methanol and PBST (0.1% Triton). To block endogenous peroxidase, the slices were treated with 10% normal goat serum (NGS) in 0.1% PBST. Sections were incubated over night at 4°C with a primary antibody against PAR (Abcam #ab14460; diluted 1:200), as a marker for PARP activity. For detection, an appropriate biotinylated secondary antibody (mouse, diluted 1:150) and an ABC kit (Vector, Burlingame, CA, United States), including avidin and biotinylated horseradish peroxidase, were used according to the manufacturer’s instructions (dilution 1:150 each). For chromogenic detection, the slices were finally placed for 2 min in a DAB-solution that contained phosphate buffer (0.1 M), glucose (20%), NH_4_Cl (0.4%), nickel ammonium sulfate (1%), glucoseoxidase, and DAB. Sections were cover slipped with Aquatex (Aquatex, Merck, Darmstadt) and analyzed using a BX61 microscope (Olympus, Hamburg, Germany).

### RNA Isolation and Reverse Transcription-PCR

For RNA isolation, apical and medial turns of the organ of Corti from 28-day-old mice were dissected and placed on a coverslip. Hensen’s and Claudius’ cells were removed with cleaning pipettes. 30–60 OHCs were harvested with micropipettes under flow of Tris-Cl solution (0.7 ml/min). Subsequently, outer pillar cells were removed and 30 IHCs were harvested with their adjacent supporting cells (inner phalangeal and border cells). For SG dissection, the cochleae were opened, the stria vascularis and organ of Corti were removed, and the modiolus with SG was used. For cochlea dissection, the bone was removed and the whole remaining tissue was used. The used tissue was frozen in liquid nitrogen. For each age and species, experiments were repeated at least three times.

For isolated OHCs and IHCs, cell lysis and reverse transcription into cDNA was started directly by sampling from the micropipette into the tube (final volume 20 μl). Always, for RT-PCR 5 μl cDNA was used. The first PCR reaction of the nested PCR approach contained enough PCR product for detection in the second PCR reaction. From SG of four cochleae, about 270 ng RNA was isolated. From two mice, total cochleae about 480 ng was isolated. The amount and quality of the RNA were analyzed photometrically. The resulting 260/280 ratio of about 2 (regularly 1.9–2.1 in our hands) was considered as pure.

The PCR program, according to the manufacturer’s instructions, included an initial activation step at 95°C for 3 min, followed by 35 cycles of a 30 s denaturing step at 95°C, a 30 s combined annealing/extension step at 55°C, and 25 s at 72°C; ending with 5 min at 72°C. For nested RT-PCR, the PCR program included an initial activation step at 95°C for 3 min, followed by 35 cycles of a 30 s denaturing step at 95°C, a 30 s combined annealing/extension step at 58°C, and 30 s at 72°C; ending with 5 min at 72°C. All PCR fragments were extracted (QIAGEN Gel Extraction Kit) and sequenced to confirm product specificity.

### Data Analysis

#### ABR Fine-Structure Analysis

ABR functions were analyzed for consecutive amplitude deflections (waves), each wave consisting of a starting negative peak and the following positive peak. Peak amplitudes of noise-burst, stimulus-evoked ABR (noise-ABR) wave I were extracted with customized computer programs, as previously described (Rüttiger et al., 2013). ABR peak-to-peak (wave) amplitude input–output (I/O) growth functions were constructed for each individual ear and increasing stimulus levels with reference to the ABR thresholds using Excel (Microsoft Excel 2016, Redmond, United States).

#### Calculation of Hearing Loss Over Age

For the calculation of DPOAE amplitude loss over age (in dB), the mean value between f1 = 30–45 dB SPL (f1 = 5.6 kHz) or between f1 = 0–65 dB SPL (f1 = 11.3 kHz) was calculated. For the calculation of ABR wave amplitude loss over age (in μV), the mean value between 20 and 65 dB SPL stimulation was calculated. The means of middle-aged and old animals were each normalized to the mean of the young animals.

#### PAR Quantification

The intensity of PAR staining (as a surrogate marker for PARP activity) was quantified by asking six “blinded” volunteers to choose the darker staining of a GC-A WT and KO pair of either IHC, OHC, or SG. This procedure was repeated for *n* = 3 mice with both ears, taking pictures of at least two slices on each slide on both basal and midbasal turns. The pictures were shown in direct comparison on PowerPoint slides (Microsoft PowerPoint 2016, Redmond, United States), with arrows marking the cell nuclei of interest. During analysis, the judgment of the volunteers was evaluated by counting the number of cases for which they choose the WT as exhibiting a darker staining in the nuclei, in comparison to the KO and a relation [(*n*_cases with judgment WT darker_/n_all_)^∗^1] was calculated.

### Statistical Analysis

Results for ABR thresholds, DPOAE thresholds and ABR fine structure analysis from the two individual ears of each animal were averaged and the statistical analysis run based on the number of animals. Statistical significance of PARP activity was tested with a *z*-test to compare the mean of the decisions against chance level (0.5). Unless otherwise stated, all data are presented as group mean, with standard error of the mean (SEM). Differences of the means were compared for statistical significance either by a Student’s *t-*test, two-way ANOVA, or regression tests using Excel (Microsoft Excel 2016, Redmond, United States), or GraphPad Prism 5.01 (La Jolla, CA, United States). Two-way ANOVA tests were followed by multiple comparison tests with correction for type 1 error after Bonferroni. The chosen statistical significance level was α = 0.05, and resulting *p-*values are reported in the legends using (^∗^)*P* < 0.1; ^∗^*P* < 0.05; ^∗∗^*P* < 0.01; ^∗∗∗^*P* < 0.001; n.s., not significant.

## Results

### GC-A, ANP, BNP, and PDE9a Expression in Cochlear Cells

**FIGURE 1:**
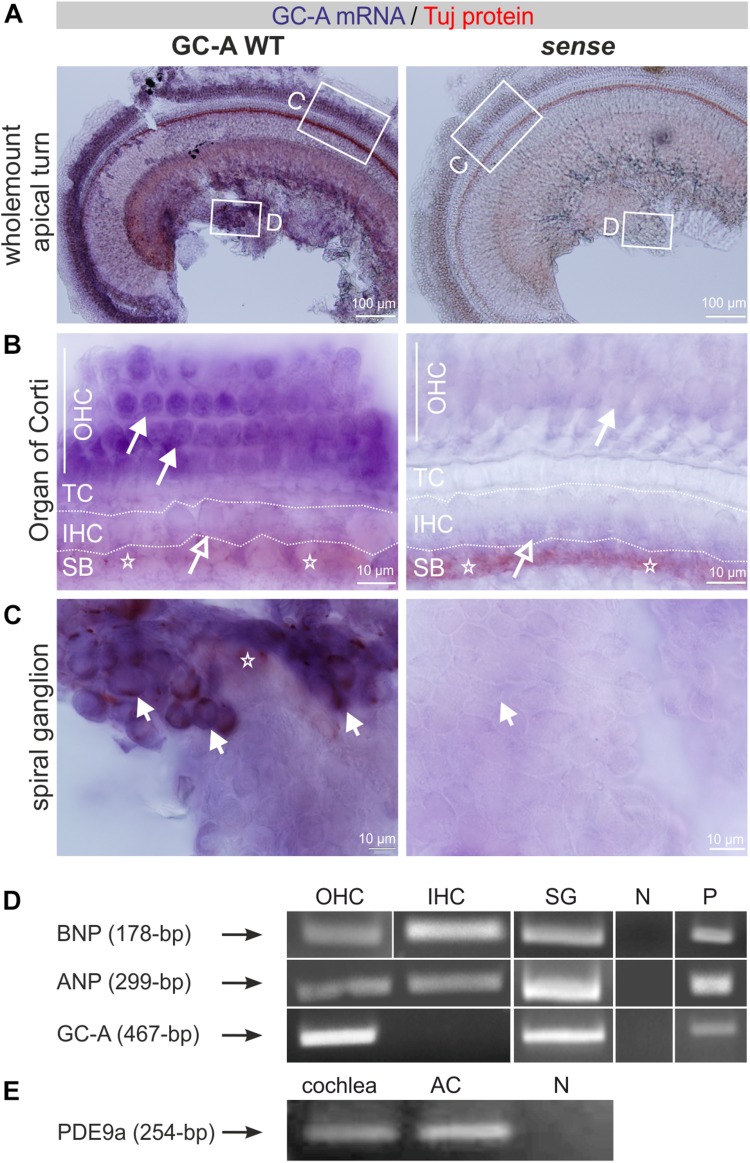
Expression of GC-A, its ligands, and PDE9a in the cochlea. **(A)** GC-A mRNA and Tuj-1 protein staining in GC-A WT antisense and sense whole-mount cochlear preparations. Scale bars: 100 μm. **(B)** GC-A mRNA staining was shown in GC-A WT OHC (closed arrows), but not IHC (open arrows). Tuj-1 staining (stars) in the spiral bundle (SB) in both WT and KO. TC = tunnel of Corti. Scale bars: 10 μm. **(C)** GC-A mRNA staining was shown in GC-A WT SGN (short arrows) but not in GC-A KO. Tuj-1 staining (stars) in the SGN. Scale bars: 10 μm. **(D)** Reverse transcription-PCR of WT IHC and OHC cDNA shows a fragment of ANP (299-bp) and BNP (178-bp) expressed in isolated OHCs, IHCs, and SG. GC-A at 467-bp was only found in OHCs, but not IHCs. **(E)** PDE9a was expressed in both cochlear and auditory-cortex (AC) tissue samples. Abbreviations: N, negative control; P, positive control (heart).

To study differential compartment- or cell-specific GC-A expression, GC-A-specific riboprobes were generated (see section “Materials and Methods”) and colocalized on free-floating whole-mount cochleae with mouse monoclonal neuron-specific class III β-tubulin antibody. Tuj-1 is a marker of neural cochlear fibers (Molea et al., 1999; Liu et al., 2009). Double detection of mRNA and protein in whole-mount cochlear preparations was performed as previously described for analysis of brain vibratome sections (Singer et al., 2014; see section “Materials and Methods”). Whole-mount cochlear preparations were dissected separately for apical, medial, and midbasal cochlear regions and mounted before visualization. We observed strong GC-A staining in OHCs (Figure 1A) and no staining in sense controls (Figure 1A). With higher magnification, GC-A staining in OHCs (Figure 1B, closed arrow) and absence of GC-A in IHCs (Figure 1B, open arrow), close to Tuj-1-positive afferent terminals at the IHC level (Figure 1B, stars), became evident. Many, but not all, SGNs also strongly expressed GC-A (Figure 1C, short arrows).

With the aim of strengthening understanding of the regulatory role of GC-A and its ligands in possible hair-cell-specific effects, we investigated ANP, BNP, and GC-A mRNA expression in isolated hair cells. IHCs and OHCs were dissected from adult mice as described in section “Materials and Methods,” and mRNA was isolated as described (Engel et al., 2006; Möhrle et al., 2017). All PCR fragments were extracted and sequenced as described in the methods to confirm product specificity. As shown by mRNA analyses, nested primers amplified a GC-A-specific fragment in OHCs and SG but not in IHCs, while ANP and BNP mRNA was detected in IHCs, OHCs, and SG (Figure 1D).

Because the cGMP-degrading enzyme PDE9a might be a target accessible to drug influence to increase cGMP pools that are predominantly controlled by ANP/GC-A (Lee et al., 2015), we also explicitly searched for PDE9a expression in the cochlea. PDE9a was expressed in the cochlea and in the auditory cortex (AC) (Figure 1E).

### Accelerated Progression of Age-Related Hearing Loss in GC-A KO Mice

**FIGURE 2:**
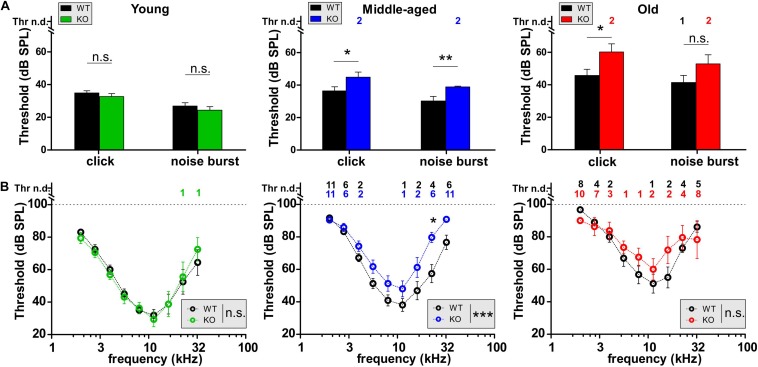
Auditory brainstem responses (ABRs) in GC-A WT and KO mice over age. **(A,B)** Hearing thresholds of WT and GC-A KO littermates assessed from auditory brainstem response (ABR) potentials in response to low-frequency-containing (click), high-frequency containing (noise burst), and pure-tone frequency-specific auditory stimuli. **(A)** Click-evoked ABR thresholds were not affected in young GC-A KO animals [green, unpaired two-tailed student’s *t*-test: *t*(30) = 0.9870, *P* = 0.3315, *n* = 8/16 mice/ears each], but elevated in middle-aged [blue, unpaired two-tailed student’s *t*-test: *t*(100) = 2.125, *P* = 0.0361, WT *n* = 27/54 mice/ears; KO *n* = 48/24 mice/ears] and old [red, unpaired two-tailed student’s *t*-test: *t*(47) = 2.350, *P* = 0.0230, WT *n* = 14/28 mice/ears; KO *n* = 11/21 mice/ears] GC-A KOs compared to WTs. Also, noise-burst evoked ABR thresholds were elevated in middle-aged GC-A KOs [blue, unpaired two-tailed student’s *t*-test: *t*(100) = 2.890, *P* = 0.0047, WT *n* = 27/54 mice/ears; KO *n* = 24/48 mice/ears]. Young and old animals did not show differences in noise-burst evoked ABR thresholds [young: unpaired two-tailed student’s *t*-test: *t*(30) = 0.8649, *P* = 0.3940, *n* = 8/16 mice/ears each; old: unpaired two-tailed student’s *t*-test: *t*(47) = 1.648, *P* = 0.1509, WT *n* = 14/28 mice/ears; KO *n* = 10/21 mice/ears]. **(B)** With pure-tone frequency-specific stimuli in the range between 2 and 32 kHz, middle-aged GC-A KOs (blue) had increased ABR thresholds compared to WTs [two-way ANOVA: *F*(1,8) = 26.54, *P* < 0.0001, WT *n* = 27/27 mice/ears; KO *n* = 23/23 mice/ears]. Young and old animals did not show differences [young: two-way ANOVA: *F*(1,126) = 0.00, *P* = 0.9781, *n* = 8/8 mice/ears each; old: two-way ANOVA: *F*(1,142) = 1.43 *P* = 0.2235, WT *n* = 14/14 mice/ears; KO *n* = 10/10 mice/ears]. Thr n.d. = Threshold not detectable. Mean ± SEM. ^∗^*P* < 0.05; ^∗∗^*P* < 0.01; ^∗∗∗^*P* < 0.001.

To study the effect of GC-A gene disruption on hearing, we first compared the hearing thresholds of age-matched young (2–4 months), middle-aged (7–12 months), and old (16–18 months) unexposed GC-A WT and KO mice. The ABR evoked by low frequency-containing (click), high frequency-containing (noise-burst), and pure tone frequency-specific auditory stimuli were tested as described (Jaumann et al., 2012; Möhrle et al., 2016). As shown in Figure 2A, young GC-A WT and KO mice did not differ in hearing thresholds for click- or for noise-burst stimuli (Figure 2A, left panel and Table 2A). An elevation in hearing threshold to pure tone auditory stimuli > 22 kHz in GC-A KO mice compared to GC-A WT mice is apparent (Figure 2B, left panel), but this difference did not reach statistical significance. In contrast, both middle-aged (Figure 2A, middle panel) and old GC-A animals (Figure 2A, right panel) exhibited elevated thresholds for click, noise-burst, and frequency-specific stimuli, and the changes were most pronounced at middle ages. The typically-occurring, profound age-dependent elevation in hearing thresholds in the last third of life (Figures 2A,B and Table 1) partially abolished the differences in ABR thresholds between old GC-A WT and KO mice. This was confirmed when frequency-specific ABR thresholds were compared in GC-A WT and KO mice. A threshold elevation became particularly evident in middle-aged GC-A KO mice (Figure 2B, middle panel), but not in old GC-A KO mice compared to GC-A WT mice (Figure 2B, right panel).

**TABLE 1:** Primer sequences and information used for PCR.

	**Position and length**	**Forward**	**Reverse**
ANP	Accession number BC089615, position 194-609, 416-bp	5′-GTA CAG TGC GGT GTC CAA CA-3′ (Zhang et al., 2017)	5′-GCT CAA GCA GAA TCG ACT GC-3′ (Nie et al., 2018)
ANP nested	Position 204-502, 299-bp	5′-TTC AAG AAC CTG CTA GAC CAC C-3′ Self-designed	5′-CCA ATC CTG TCA ATC CTA CCC C-3′ Self-designed
BNP	Accession number BC061165, position 202-424, 222-bp	5′-AAG CTG CTG GAG CTG ATA AGA-3′ (Kuhn et al., 2009)	5′-GTT ACA GCC CAA ACG ACT GAC-3′ (Kuhn et al., 2009)
BNP nested	Position 224-401, 178-bp	5′-GAA AAG TCG GAG GAA ATG GCC C-3′ Self-designed	5′-ATC CGA TCC GGT CTA TCT TGT GC-3′ Self-designed
GC-A	Accession number BC110659, position 1927-2599, 702-bp	5′-TGT GAA ACG TGT GAA CCG GA-3′ Self-designed	5′-AGG CGG ATC GTT GAA AGG G-3′ Self-designed
GC-A nested	Position 1998-2464, 467-bp	5′-TGT GCA GAA TGA GCA CTT GAC C-3′ Self-designed	5′-CCA AAC CTT CCA CAT AGA AGA CCC-3′ Self-designed
PDE9a	Accession number NM_008804, position 199-452, 254-bp	5′-ACC ACC ATC TCC CTT TTA ACC-3′ Self-designed	5′-AGT CCT TCC AAT TCC ACC C-3′ Self-designed

**TABLE 2:** Table of statistics.

****(A)** Click, noise-burst, and frequency-specific ABR thresholds in GC-A WT and KO mice as a function of age.**			

	**Young**	**Middle-aged**	**Old**
Click	Unpaired two-tailed student’s *t*-test: t(30) = 0.9870 *P* = 0.3315, *n* = 8/16 mice/ears each	Unpaired two-tailed student’s *t*-test: *t*(100) = 2.125 *P* = 0.0361, WT *n* = 27/54 mice/ears; KO *n* = 48/24 mice/ears	Unpaired two-tailed student’s *t*-test: *t*(47) = 2.350 *P* = 0.0230, WT *n* = 14/28 mice/ears; KO *n* = 11/21 mice/ears
Noise burst	Unpaired two-tailed student’s *t*-test: *t*(30) = 0.8649 *P* = 0.3940, *n* = 8/16 mice/ears each	Unpaired two-tailed student’s *t*-test: *t*(100) = 2.890 *P* = 0.0047, WT *n* = 27/54 mice/ears; KO *n* = 24/48 mice/ears	Unpaired two-tailed student’s *t*-test: *t*(47) = 1.648 *P* = 0.1509, WT *n* = 14/28 mice/ears; KO *n* = 10/21 mice/ears
Frequency	Two-way ANOVA: *F*(1,126) = 0.00 *P* = 0.9781, *n* = 8/8 mice/ears each	Two-way ANOVA: *F*(1,8) = 26.54 *P* < 0.0001, WT *n* = 27/27 mice/ears; KO *n* = 23/23 mice/ears	Two-way ANOVA: *F*(1,142) = 1.43 *P* = 0.2235, WT *n* = 14/14 mice/ears; KO *n* = 10/10 mice/ears

**(B) Thresholds and input/output function of DPOAEs at different f1 frequencies in GC-A WT and KO mice as a function of age.**			

	**Young**	**Middle-aged**	**Old**

Threshold	Two-way ANOVA, *F*(1,84) = 0.04, *P* = 0.8510, *n* = 8/16 mice/ears each	Two-way ANOVA, *F*(1,132) = 1.54, *P* = 0.2157, WT *n* = 6/12 mice/ears KO *n* = 8/16 mice/ears	Two-way ANOVA, *F*(1,247) = 0.52, *P* = 0.4697, WT *n* = 14/27 mice/ears KO *n* = 10/20 mice/ears

5.6 kHz	Two-way ANOVA, *F*(1,382) = 0.17, *P* = 0.679, *n* = 8/16 mice/ears each	Two-way ANOVA, *F*(1,577) = 0.00, *P* = 0.9879, WT *n* = 10/20 mice/ears KO *n* = 14/28 mice/ears	Two-way ANOVA, *F*(1,382) = 0.59, *P* = 0.4439, *n* = 8/16 mice/ears each
11.3 kHz	Two-way ANOVA, *F*(1,382) = 4.40, *P* = 0.0367, *n* = 8/16 mice/ears each;	Two-way ANOVA, *F*(1,577) = 3.34, *P* = 0.0681, WT *n* = 10/20 mice/ears KO *n* = 14/28 mice/ears	Two-way ANOVA, *F*(1,382) = 14.01, *P* = 0.0002, *n* = 8/16 mice/ears each

**(C) OHC function after acoustic trauma in young GC-A WT and KO mice.**		

**Delta ABR threshold**		**Delta DPOAE threshold**

Two-way ANOVA, *F*(1,44) = 3.11, *P* = 0.0845, WT *n* = 3/3 mice/ears KO *n* = 4/4 mice/ears		Two-way ANOVA, *F*(1,84) = 1.43, *P* = 0.2344, WT *n* = 3/6 mice/ears KO *n* = 4/8 mice/ears

	**5.6 kHz**	**11.3 kHz**

Post I/O	Two-way ANOVA, *F*(1,147) = 2.31, *P* > 0.05, WT *n* = 3/6 mice/ears KO *n* = 4/8 mice/ears	Two-way ANOVA, *F*(1,147) = 1.46, *P* > 0.05, WT *n* = 3/6 mice/ears KO *n* = 4/8 mice/ears

	**5.6 kHz**	**11.3 kHz**

Delta I/O	Two-way ANOVA, *F*(1,121) = 0.03, *P* = 0.8693, WT *n* = 3/6 mice/ears KO *n* = 4/7 mice/ears	Two-way ANOVA, *F*(1,180) = 6.06, *P* = 0.0148, WT *n* = 3/6 mice/ears KO *n* = 4/8 mice/ears
Regression	*t*(183) = 0.226, *P* = 0.98, WT *n* = 85 KO *n* = 102	*t*(69) = 0.027, *P* = 0.98, WT *n* = 28 KO *n* = 45

**(D) Supra-threshold ABR wave I and IV amplitudes in GC-A WT and KO mice as a function of age and before and after acoustic trauma.**			

	**Young**	**Middle-aged**	**Old**

ABR wave I	Two-way ANOVA, *F*(1,374) = 10.57, *P* = 0.0013, *n* = 8/16 mice/ears each	Two-way ANOVA, *F*(1,247) = 5.38, *P* = 0.0212, WT *n* = 6/12 mice/ears KO *n* = 5/10 mice/ears	Two-way ANOVA, *F*(1,255) = 82.55, *P* < 0.0001, WT *n* = 7/14 mice/ears KO *n* = 5/10 mice/ears
ABR wave IV	Two-way ANOVA, *F*(1,362) = 0.00, *P* = 0.9568, *n* = 8/16 mice/ears each	Two-way ANOVA, *F*(1,462) = 32.21, *P* < 0.0001, WT *n* = 11/21 mice/ears KO *n* = 10/20 mice/ears	Two-way ANOVA, *F*(1,269) = 43.28, *P* < 0.0001, WT *n* = 7/14 mice/ears each
ABR wave I post acoustic trauma	Two-way ANOVA, *F*(1,117) = 36.46, *P* < 0.0001, WT *n* = 3/6 mice/ears KO *n* = 4/8 mice/ears	Two-way ANOVA, *F*(1,105) = 4.84, *P* = 0.0300, *n* = 5/10 mice/ears each	
ABR wave IV post acoustic trauma	Two-way ANOVA, *F*(1,113) = 17.20, *P* < 0.0001, WT *n* = 3/6 mice/ears KO *n* = 4/8 mice/ears	Two-way ANOVA, *F*(1,108) = 17.58, *P* < 0.0001, WT *n* = 5/10 mice/ears KO *n* = 6/12 mice/ears	

**(E) IHC ribbon numbers in GC-A WT and KO mice as a function of age and before and after acoustic trauma (AT).**			

	**Young**	**Middle-aged**	**Old**

Basal turn	Two-way ANOVA, Genotype: *F*(1,20) = 65.9, *P* < 0.0001, *n* = 6/3 samples/mice each; AT: *F*(1,20) = 185.69, *P* < 0.0001, *n* = 6/3 samples/mice each, *post hoc* test: sham WT vs. sham KO *P* < 0.001, sham KO vs. AT WT *P* < 0.001, AT WT vs. AT KO *P* < 0.0001	Two-way ANOVA, Genotype: F(1,23) = 15.40, *P* = 0.0007, WT *n* = 8/4 samples/mice KO *n* = 7/4 samples/mice; AT: *F*(1,23) = 14.96, *P* = *0.0008*, WT *n* = 6/4 samples/mice KO *n* = 6/4 samples/mice, *post hoc* test: sham WT vs. sham KO *P* < 0.05, sham KO vs. AT WT *P* > 0.05, AT WT vs. AT KO *P* > 0.05	Unpaired two-tailed student’s *t*-test, *t*(5) = 5.811 *P* < 0.0002, *n* = 6/3 samples/mice each
Midbasal turn	Two-way ANOVA, Genotype: *F*(1,21) = 74.62, *P* < 0.0001, *n* = 6/3 samples/mice each; AT: *F*(1,21) = 41.97, *P* < 0.0001, *n* = 6/3 samples/mice each, *post hoc* test: sham WT vs. sham KO *P* < 0.01, sham KO vs. AT WT *P* > 0.05, AT WT vs. AT KO *P* < 0.0001	Two-way ANOVA, Genotype: *F*(1,25) = 47.12, *P* < 0.0001 WT *n* = 7/4 samples/mice KO *n* = 6/4 samples/mice; AT: *F*(1,25) = 37.21, *P* < 0.0001, Interaction: *F*(1,25) = 6.926, *P* = 0.0143, WT *n* = 8/4 samples/mice KO *n* = 7/4 samples/mice, *post hoc* test: sham WT vs. sham KO *P* < 0.0001, sham KO vs. AT WT *P* > 0.05, AT WT vs. AT KO *P* > 0.05	Unpaired two-tailed student’s *t*-test, *t*(10) = 5.580 *P* < 0.0002, *n* = 6/3 samples/mice each
Apical turn	Two-way ANOVA, Genotype: *F*(1,20) = 19.49, *P* = 0.0003; *n* = 6/3 samples/mice each, AT: *F*(1,20) = 6.307, *P* = 0.0207, *n* = 6/3 samples/mice each, Interaction: *F*(1,20) = 7.510, *P* = 0.0126, *post hoc* test: sham WT vs. sham KO *P* < 0.01, sham KO vs. AT WT *P* < 0.001, AT WT vs. AT KO *P* > 0.05	Two-way ANOVA, Genotype: *F*(1,24) = 11.41, *P* = 0.0025 WT *n* = 7/4 samples/mice KO *n* = 6/4 samples/mice; *F*(1,24) = 2.740, *P* = 0.1109 WT *n* = 8/4 samples/mice KO *n* = 7/4 samples/mice, *post hoc* test: sham WT vs. sham KO *P* > 0.05, sham KO vs. AT WT *P* < 0.01, AT WT vs. AT KO *P* > 0.05	Unpaired two-tailed student’s *t*-test, *t*(10) = 2.789 *P* = 0.0192, *n* = 6/3 samples/mice each

**(F) Aging progress in OHC function and auditory nerve responses.**			

	**DPOAE 5.6 kHz**	**DPOAE 11.3 kHz**	**Noise burst ABR wave I**

Loss of amplitude	Two-way ANOVA, *F*(1,106) = 0.01, *P* = 0.928, *n* = 8–14/16–28 mice/ears each	Two-way ANOVA, *F*(1,106) = 0.00, *P* = 0.951, *n* = 8–14/16–28 mice/ears each	Two-way ANOVA, *F*(1,71) = 4.72, *P* = 0.0033, *n* = 5–8/10–16 mice/ears each

**TABLE 3:** Antibodies for immunostaining.

	**Host organism**	**Dilution**	**Company**
Prestin	Rabbit	1:3000	Squarix, Berlin, Germany #976102#5
KCNQ4	Mouse	1:50	Stress marq, British Columbia, United Kingdom SMC-309D
CtBP2/RIBEYE	Rabbit	1:1500	American Research Products, Waltham, United States #10-P1554
Tuj1	Mouse	1:500	BioLegend/Biozol, Eching, Germany #801201
PAR	Chicken	1:200	Abcam, Cambridge, United Kingdom #ab14460
Digoxigenin	Sheep	1:750	Anti-Dig-AP, Roche, Germany, 11093274910
Biotinylated IgG	Goat	1:500/1:150	Vector Laboratories, BA-1000
Cy3	Goat	1:1500	Jackson Immuno Research Laboratories, West Grove PA, United States
Alexa488	Goat	1:500	Invitrogen Molecular Probes, Paisley, United Kingdom

It was shown that during the last two-thirds of life, GC-A KO mice developed elevated hearing thresholds relative to GC-A WT mice.

### GC-A KO Mice Exhibit Early Dysfunction of OHCs Independent of Age

**FIGURE 3:**
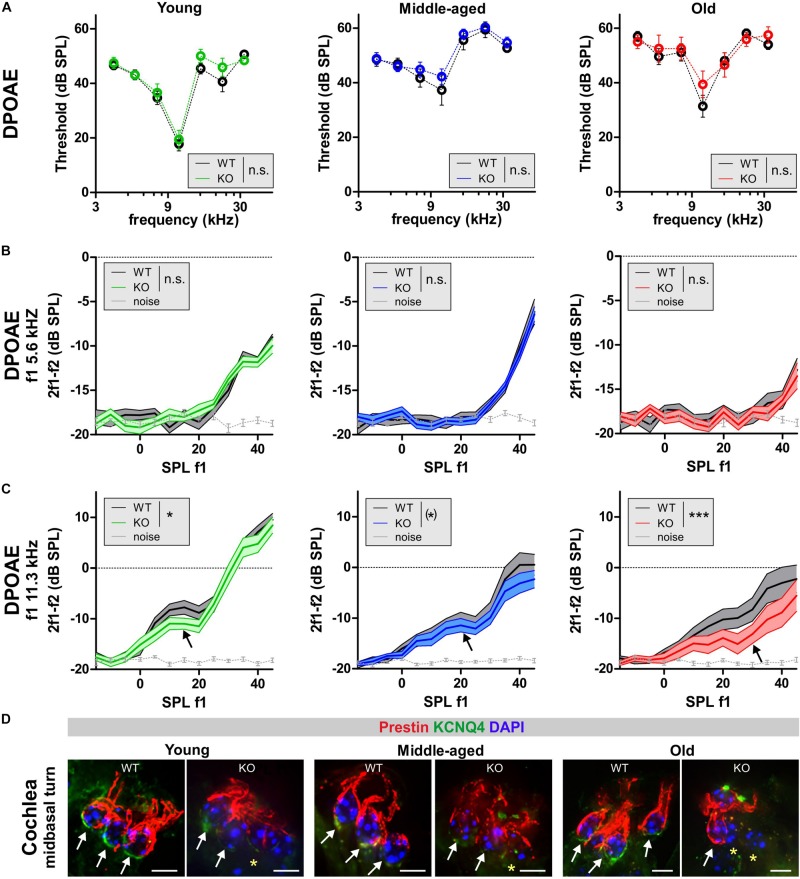
DPOAEs and expression of KCNQ4 in GC-A WT and GC-A KO mice at different ages. **(A–C)** OHC function assessed from distortion-product otoacoustic emissions (DPOAEs) that are generated by electromechanical responses of OHCs. **(A)** Frequency-specific thresholds of DPOAE signals for pure-tone sounds from f2 = 4–32 kHz did not differ between GC-A KO and WT mice [young: two-way ANOVA, *F*(1,84) = 0.04, *P* = 0.8510, *n* = 8/16 mice/ears each; middle-aged: two-way ANOVA, *F*(1,132) = 1.54, *P* = 0.2157, WT *n* = 6/12 mice/ears KO *n* = 8/16 mice/ears; old: two-way ANOVA, *F*(1,247) = 0.52, *P* = 0.4697, WT *n* = 14/27 mice/ears KO *n* = 10/20 mice/ears]. **(B)** DPOAE growth function in response to pure-tone sounds at f1 = 5.6 kHz were similar between GC-A KO and WT mice in all three age groups [young: two-way ANOVA, *F*(1,382) = 0.17, *P* = 0.679, *n* = 8/16 mice/ears each; middle-aged: two-way ANOVA, *F*(1,577) = 0.00, *P* = 0.9879, WT *n* = 10/20 mice/ears KO *n* = 14/28 mice/ears; old: two-way ANOVA, *F*(1,382) = 0.59, *P* = 0.4439, *n* = 8/16 mice/ears each]. **(C)** DPOAE growth function in response to pure-tone sounds at f1 = 11.3 kHz were reduced in GC-A KO compared to WT mice in all three age groups [young: two-way ANOVA, *F*(1,382) = 4.40, *P* = 0.0367, *n* = 8/16 mice/ears each; middle-aged: two-way ANOVA, *F*(1,577) = 3.34, *P* = 0.0681, WT *n* = 10/20 mice/ears KO *n* = 14/28 mice/ears; old: two-way ANOVA, *F*(1,382) = 14.01, *P* = 0.0002, *n* = 8/16 mice/ears each]. Mean ± SEM. ^∗^*P* < 0.1; ^∗^*P* < 0.05; ^∗∗∗^*P* < 0.001. **(D)** The intactness of the OHC phenotype was investigated by immunohistochemical staining for KCNQ4 (green) and the motor protein prestin (red) as markers for OHC viability and their capacity for electromechanical responses. Staining for KCNQ4 in cochlear OHCs of GC-A mice was reduced in OHCs (upper panel) in comparison to WT mice, while prestin seems to be only slightly reduced due to degeneration of membrane. Nuclei were stained with 4’,6-diamidin-2-phenylindol (DAPI, blue). Yellow asterisk shows absence of KCNQ4 in OHCs. Scale bars: 5 μm.

To assign the hearing threshold elevation in GC-A KO mice to specific cochlear compartments, we first analyzed electromotile properties of OHCs that form the basis of sound-evoked neural potentials at threshold (Marcon and Patuzzi, 2008). Electromotile properties of OHCs can be assessed by recording ear-canal sound-pressure changes induced by DPOAEs (Shera and Guinan, 1999) that are specifically generated by electromechanical responses of OHCs (El-Badry and McFadden, 2007; Rüttiger et al., 2017). Frequency-specific thresholds of DPOAE signals from amplitude I/O functions were analyzed by presenting pure-tone sounds from f2 = 4–32 kHz and increasing sound level (L2 = −10 to 45 dB SPL) in young, middle-aged, and old GC-A WT and GC-A KO mice (Figure 3 and Table 2B). Despite differences in ABR thresholds in response to click, noise-burst, and frequency-specific stimuli (Figure 2), DPOAE thresholds were similar between WT and GC-A KO mice for all ages tested (Figure 3A). However, when the I/O functions of DPOAEs for f1 = 5.6 and 11.3 kHz were compared between GC-A WT and KO mice at different ages, it became evident that DPOAE I/O responses to f1 = 5.6 kHz remained similar between GC-A KO and WT mice across the different age groups (Figure 3B). In contrast, OHC-specific responses at higher frequencies (f1 = 11.3 kHz) were already reduced in young GC-A KO mice (Figure 3C). Interestingly, the difference in DPOAE I/O responses between GC-A KO mice and WT mice remained constant throughout all ages, in line with a typically occurring, age-dependent hearing loss that progresses independently of GC-A signaling in the last third of life.

Previously, increased cGMP levels have been shown to protect against noise-induced loss of the membranous potassium voltage-gated channel subfamily KQT member 4 (KCNQ4) in OHCs. KCNQ4 is the voltage-dependent K^+^ channel that maintains the OHC resting potential and is vital for OHC survival (Marcotti and Kros, 1999). Therefore, we further investigated the impact of GC-A gene disruption on the expression pattern of KCNQ4 in OHCs from young, middle-aged, and old mice. Using high-resolution confocal microscopy, KCNQ4 was co-stained with the OHC motor protein prestin, which is encoded by the *Slc26a5* gene and responsible for the electromechanical properties of OHCs (Zheng et al., 2000) (Figure 3). KCNQ4 surface expression at the base of OHCs was reduced in high-frequency cochlear regions of GC-A KO mice of all ages compared to GC-A WT mice (Figure 3D, mid-basal turn, yellow stars), as shown by *n* = 3 independent repetitions. In contrast, membrane staining of the OHC motor protein prestin was preserved in the lateral walls of OHCs across age, although the intensity of prestin staining in OHCs from GC-A KO mice appeared to be slightly reduced in aged animals (Figure 3D) because of degeneration of cell membrane in which prestin is placed.

GC-A KO mice already showed impaired OHC function compared to GC-A WT mice at a young age.

### GC-A KO Mice Exhibit Early Dysfunction of OHCs Independent of Acoustic Trauma

**FIGURE 4:**
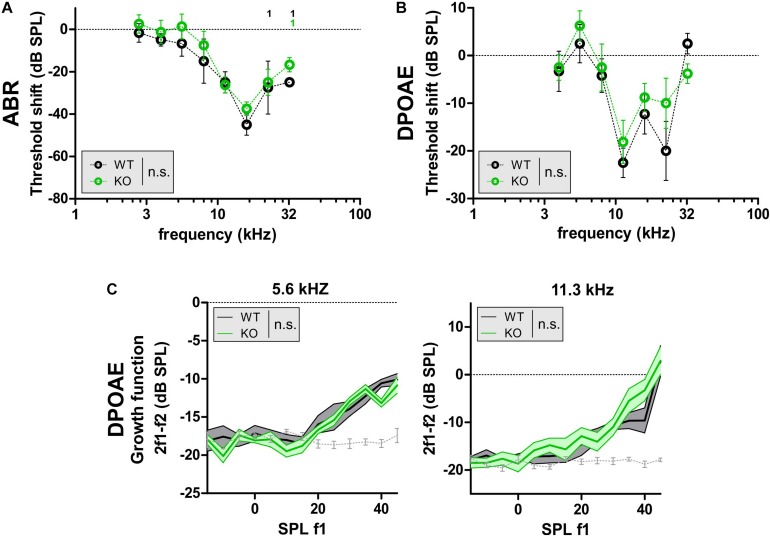
DPOAE signals in GC-A WT and GC-A KO mice after acoustic trauma. **(A–C)** ABR threshold and OHC function, assessed by DPOAE measurements 7 days after acoustic trauma induction in young GC-A WT and KO mice. **(A,B)** Shift of frequency-specific ABR thresholds and DPOAE thresholds, when compared before and 7 days after acoustic trauma, did not show differences between WT and KO mice [f-ABR: two-way ANOVA, *F*(1,44) = 3.11, *P* = 0.0845, WT *n* = 3/3 mice/ears KO *n* = 4/4 mice/ears; DPOAE: two-way ANOVA, *F*(1,84) = 1.43, *P* = 0.2344, WT *n* = 3/6 mice/ears KO *n* = 4/8 mice/ears]. **(C)** DPOAE signal growth function in response to pure tones at f1 = 5.6 and 11.3 kHz were similar between GC-A KO and WT mice after acoustic trauma [5 kHz: two-way ANOVA, *F*(1,147) = 2.31, *P* > 0.05, WT *n* = 3/6 mice/ears KO *n* = 4/8 mice/ears; 11 kHz: two-way ANOVA, *F*(1,147) = 1.46, *P* > 0.05, WT *n* = 3/6 mice/ears KO *n* = 4/8 mice/ears]. DPOAE signal growth function in response to pure tone sounds at f1 = 22 kHz were also smaller in GC-A KO than WT mice after acoustic trauma [two-way ANOVA, *F*(1,147) = 11.51, *P* = 0.0009, WT *n* = 3/6 mice/ears KO *n* = 4/8 mice/ears].

Noise exposure is a major cause of age-dependent hearing loss, because it can induce sensory-cell degeneration, especially in the OHCs at the high-frequency end of the cochlea (Keithley, 2019). To study whether GC-A/cGMP signaling attenuates NIHL, young GC-A WT and KO mice received an AT induced by exposure to 8–16 kHz, 120 dB SPL_rms_ sound for 40 min (see section “Materials and Methods”). Hearing loss, evident through threshold shifts, was analyzed using frequency-specific ABRs 7 days after acoustic-trauma induction. Young GC-A WT and KO mice did not differ in their degree of hearing loss in response to the traumatizing noise. This was evident by comparison of frequency-specific ABRs (Figure 4A and Table 2C) and DPOAEs (Figure 4B). In addition, the threshold shift in GC-A WT and KO mice in response to AT did not differ when analyzing DPOAE I/O responses to f1 = 5.6 kHz and 11.3 kHz stimuli (Figure 4C), although the 11.3 kHz I/O functions showed stronger loss of DPOAE signal in GC-A WT mice than in KO mice (Supplementary Figure S1A). To clarify a possible alteration in the decline of OHC motility (DPOAEs) in GC-A KO mice, the regression line between the measured DPOAE signal (in dB SPL) after AT and the loss of DPOAE signal (in dB SPL) was calculated, but not found to be different between GC-A WT and KO mice with f1 = 5.6 kHz or with f1 = 11.3 kHz stimulus (Supplementary Figure S1C). This suggests that the relative loss of DPOAE I/O function after AT is comparable in GC-A WT and KO mice.

It was shown that GC-A KO mice already have deficits in OHC function in higher frequency regions at f1 = 11.3 kHz at a young age. Moreover, this GC-A-dependent loss in OHC function is not further worsened by aging or AT.

### GC-A KO Mice Exhibit Early, Age- and Acoustic Trauma-Induced Neuropathy and Synaptopathy

**FIGURE 5:**
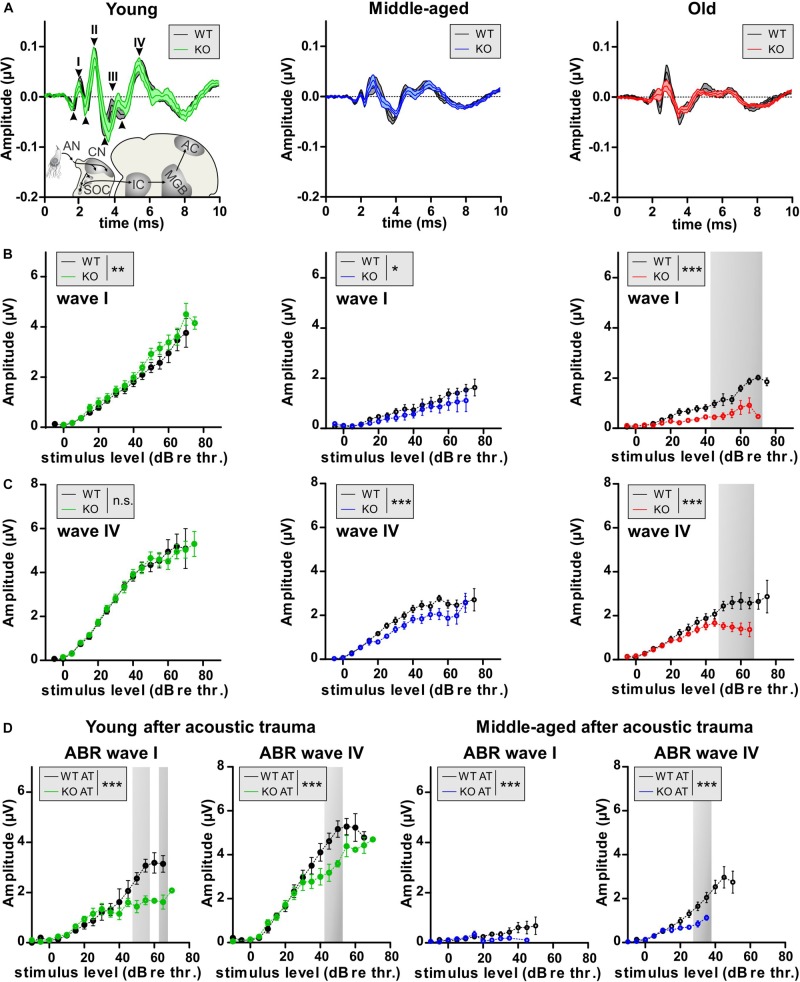
Auditory nerve and brainstem response amplitudes in GC-A WT and KO mice. **(A)** ABR waveform, indicating ABR wave I peak-to-peak amplitude 40 dB above the hearing threshold. Mean ± SEM. **(B)** The noise-burst evoked ABR wave I as a measure of the summed activity of auditory-nerve fibers assessed to investigate the effect of GC-A gene disruption on auditory-nerve responses in mice before noise exposure. Noise-burst-evoked ABR wave I amplitude growth functions were affected in GC-A KO mice (young: green, middle-aged: blue, old: red) in all three age groups compared with WT mice (all ages: black) before noise exposure [young: two-way ANOVA, *F*(1,374) = 10.57, *P* = 0.0013, *n* = 8/16 mice/ears each; middle-aged: two-way ANOVA, *F*(1,247) = 5.38, *P* = 0.0212, WT *n* = 6/12 mice/ears KO *n* = 5/10 mice/ears, old: two-way ANOVA, *F*(1,255) = 82.55, *P* < 0.0001, WT *n* = 7/14 mice/ears KO *n* = 5/10 mice/ears]. **(C)** Noise-burst-evoked ABR wave IV amplitude growth functions were decreased in middle-aged and old GC-A KO mice, but not young GC-A KO mice compared to WT mice before noise exposure [young: two-way ANOVA, *F*(1,362) = 0.00, *P* = 0.9568, *n* = 8/16 mice/ears each; middle-aged: two-way ANOVA, *F*(1,462) = 32.21, *P* < 0.0001, WT *n* = 11/21 mice/ears KO *n* = 10/20 mice/ears, old: two-way ANOVA, *F*(1,269) = 43.28, *P* < 0.0001, WT *n* = 7/14 mice/ears each]. **(D)** 7 days after acoustic trauma, noise-burst-evoked ABR wave I amplitude growth functions were more decreased in young and middle-aged GC-A KO mice than in WT mice [young: two-way ANOVA, *F*(1,117) = 36.46, *P* < 0.0001, WT *n* = 3/6 mice/ears KO *n* = 4/8 mice/ears; middle-aged: two-way ANOVA, *F*(1,105) = 4.84, *P* = 0.0300, *n* = 5/10 mice/ears each]. **(E)** ABR wave IV amplitudes were also more decreased in young and middle-aged GC-A KO mice compared to WT mice 7 days after noise exposure [young: two-way ANOVA, *F*(1,113) = 17.20, *P* < 0.0001, WT *n* = 3/6 mice/ears KO *n* = 4/8 mice/ears; middle-aged: two-way ANOVA, *F*(1,108) = 17.58, *P* < 0.0001, WT *n* = 5/10 mice/ears KO *n* = 6/12 mice/ears]. Mean ± SEM. ^∗^*P* < 0.05; ^∗∗^*P* < 0.01; ^∗∗∗^*P* < 0.001.

Aging and AT have been shown to induce auditory nerve-fiber degeneration (auditory neuropathy) related to IHC nerve terminal damage (synaptopathy) in mice, non-human primates, and humans (Gleich et al., 2016; Valero et al., 2017; Wu et al., 2019). Auditory-nerve degeneration can occur independently of OHC loss and is called hidden hearing loss (Kujawa and Liberman, 2009; Furman et al., 2013). It has been shown that elevated cGMP levels can prevent AT-induced damage of IHC nerve terminals (Jaumann et al., 2012). To investigate the impact of GC-A-induced cGMP generation on the vulnerability of pre- and postsynaptic structures of IHCs, we analyzed a possible GC-A-induced neuropathy by comparing supra-threshold ABR wave amplitudes in GC-A WT and KO mice prior to and after AT and at different ages. Supra-threshold ABR wave amplitudes change proportionally with discharge rates and the number of synchronously firing auditory nerve fibers (Johnson and Kiang, 1976), the latter defined by IHC synaptic ribbons (Buran et al., 2010). Therefore, auditory neuropathy or IHC synaptopathy is well reflected by changes in supra-threshold ABR amplitudes and IHC ribbon numbers, respectively (Kujawa and Liberman, 2009; Jaumann et al., 2012; Chumak et al., 2016; Möhrle et al., 2016). The auditory stimulus-evoked ABR wave I (Figure 5A, wave I and Table 2D) reflects the summed activity of the auditory nerve fibers (Melcher and Kiang, 1996) and is a useful functional biomarker of auditory-nerve degeneration after noise exposure (Rüttiger et al., 2017), while ABR wave IV (Figure 5A, wave I) reflects the sound-induced activity generated at the level of the inferior colliculus and lateral lemniscus (Melcher and Kiang, 1996).

The analysis of supra-threshold ABR wave I (Figure 5B) and IV (Figure 5C) revealed a reduction in ABR amplitude I and ABR amplitude IV in middle-aged and old GC-A KO mice, but not in young GC-A KO mice compared to GC-A WT mice. This indicates that unlike the effect of GC-A inactivation on OHCs (which was already apparent in young KO mice), auditory-nerve responses declined in GC-A KO mice as they aged. A slight augmentation of the ABR wave I amplitudes in young GC-A KO mice (Figure 5B) was not evident in the more centrally generated ABR wave IV, suggesting that the putatively higher auditory input from the cochlea is centrally adapted or compensated (Figure 5C).

To validate the impact of GC-A on AT-induced auditory-nerve responses, young and middle-aged GC-A WT and KO mice were exposed to 8–16 kHz broad band noise (120 dB SPL_rms_ for 40 min), and ABR wave I and IV amplitudes were analyzed 7 days post AT-induction. In young and middle-aged GC-A KO mice, the AT-induced reduction in ABR wave I and IV amplitudes was more pronounced than in WT littermates (Figure 5D).

Overall, this indicated that, unlike effects on OHCs (Figures 3, 4 and Supplementary Figure S1), GC-A gene disruption accelerated age-dependent auditory-nerve vulnerability and aggravated the effect of AT.

**FIGURE 6:**
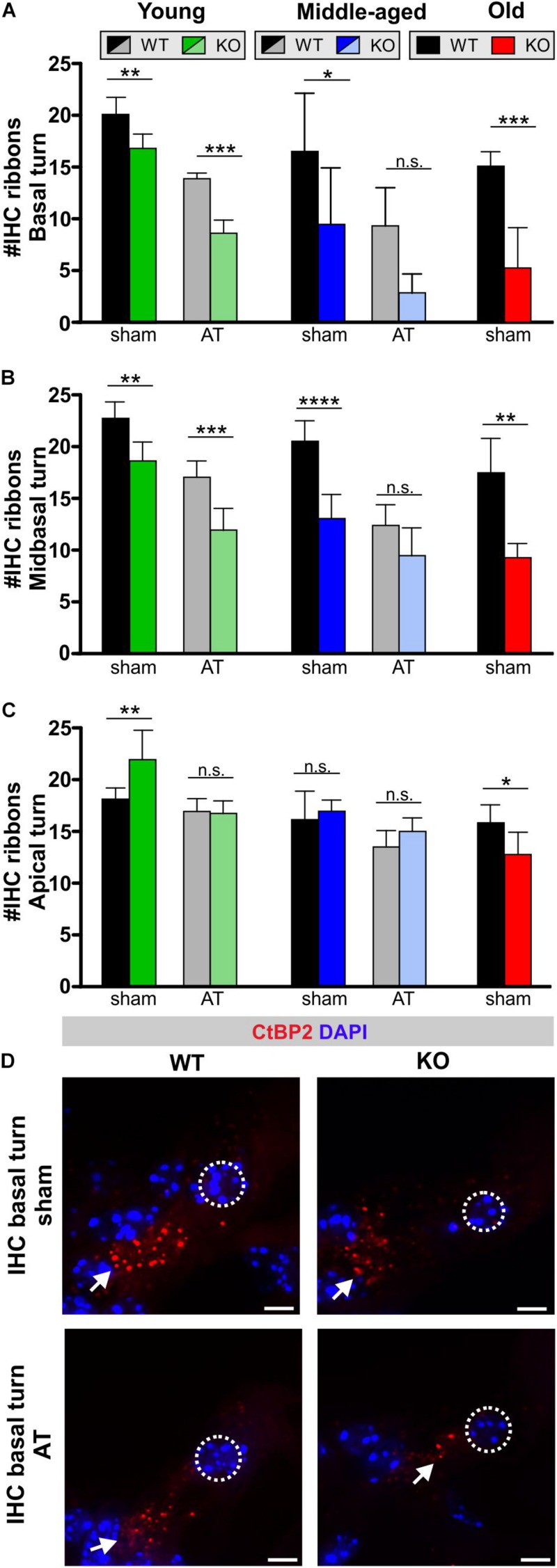
Inner hair cell ribbons in GC-A WT and KO mice 7 days after sham treatment or acoustic trauma. **(A)** Ribbon-synapse numbers of IHCs in the basal cochlear turn after sham exposure and acoustic trauma [young: two-way ANOVA, Genotype: *F*(1,20) = 65.9, *P* < 0.0001; AT: *F*(1,20) = 185.69, *P* < 0.0001, *post hoc* test: sham WT vs. sham KO *P* < 0.001, AT WT vs. AT KO *P* < 0.0001; middle-aged: two-way ANOVA, Genotype: *F*(1,23) = 15.40, *P* = *0.0007*; AT: *F*(1,23) = 14.96, *P* = *0.0008*, *post hoc* test: sham WT vs. sham KO *P* < 0.05, AT WT vs. AT KO *P* > 0.05; old: unpaired two-tailed student’s *t*-test, *t*(5) = 5.811, *P* < 0.0002]. **(B)** Ribbon-synapse numbers of IHCs in the mid-basal cochlear turn after sham exposure and acoustic trauma [young: two-way ANOVA, Genotype: *F*(1,21) = 74.62, *P* < 0.0001; AT: *F*(1,21) = 41.97, *P* < 0.0001, *post hoc* test: sham WT vs. sham KO *P* < 0.01, AT WT vs. AT KO *P* < 0.0001; middle-aged: two-way ANOVA, Genotype: *F*(1,25) = 47.12, *P* < 0.0001; AT: *F*(1,25) = 37.21, *P* < 0.0001, Interaction: *F*(1,25) = 6.926, *P* = 0.0143, *post hoc* test: sham WT vs. sham KO *P* < 0.0001, AT WT vs. AT KO *P* > 0.05; old: unpaired two-tailed student’s *t*-test, *t*(10) = 5.580, *P* < 0.0002]. **(C)** Ribbon-synapse numbers of IHCs in the apical cochlear turn after sham exposure and acoustic trauma [young: two-way ANOVA, Genotype: *F*(1,20) = 19.49, *P* = 0.0003; AT: *F*(1,20) = 6.307, *P* = 0.0207, Interaction: *F*(1,20) = 7.510, *P* = 0.0126, *post hoc* test: sham WT vs. sham KO *P* < 0.01, AT WT vs. AT KO *P* > 0.05; middle-aged: two-way ANOVA, Genotype: *F*(1,24) = 11.41, *P* = 0.0025; *F*(1,24) = 2.740, *P* = 0.1109, *post hoc* test: sham WT vs. sham KO *P* > 0.05, AT WT vs. AT KO *P* > 0.05; old: unpaired two-tailed student’s *t*-test, *t*(10) = 2.791, *P* = 0.0191]. Mean ± SD. ^∗^*P* < 0.05; ^∗∗^*P* < 0.01; ^∗∗∗^*P* < 0.001; ^****^P < 0.0001. **(D)** IHC ribbon synapses with afferent auditory neurons were stained by antibodies against CtBP2/RIBEYE. Immunopositive dots were counted to estimate the number of auditory nerve fiber synapses per IHC. The effect of GC-A gene disruption on IHC ribbon counts was analyzed in young, middle-aged and old mice. Arrows indicate a reduced number of CtBP2/RIBEYE-positive dots at the basal pole of IHCs. Nuclei were stained with DAPI (blue). Scale bars: 5 μm.

The GC-A effect on IHC synaptopathy was analyzed through staining of IHC ribbons with antibodies directed against the RIBEYE protein CtBP2. Its numbers at IHC presynaptic sides can be used as an approximate metric for the number of IHC afferent synapses (Kujawa and Liberman, 2009; Buran et al., 2010). IHC ribbon numbers in *n* = 3 or 4 animals (*n* = 6 or 8 ears) from each group were quantified in individual cochlear turns as described (Möhrle et al., 2016, 2017). The IHC ribbons in basal/midbasal turns declined with advancement in age in GC-A WT mice (Figure 6, basal turn, black bars), which has also been observed in previous studies (Kujawa and Liberman, 2009; Sergeyenko et al., 2013; Möhrle et al., 2016). The IHC ribbon numbers in basal and mid-basal cochlear turns of middle-aged and old GC-A KO mice was reduced in comparison to those in WT mice (Figures 6A,B and Table 2E). Already in young GC-A KO mice, a reduction in IHC ribbons was seen in mid-basal and basal cochlear turns (Figures 6A,B), although at that age, the IHC ribbon numbers in apical cochlear turns of GC-A KO mice were augmented (Figure 6C). Consistently, ABR wave I was not yet reduced at that age but even slightly enhanced (Figure 5B), suggesting that lower frequency cochlear regions might contribute to ABR wave I generation in response to noise-burst stimuli. In the apical cochlear turn, the GC-A KO mice still had an equal number of synaptic ribbons when compared to the WT (Figure 6C), even though the amplitudes of the ABR wave I were smaller than in the GC-A WT mice. This indicated that IHC ribbons cease to function properly before the reduction of CtBP2 protein becomes obvious from histology. Expression studies on postsynaptic markers should be considered in future experiments. In GC-A KO mice, AT led to a further loss in IHC ribbon number in these turns. For example, this is illustrated for CtBP2 immunostained IHCs in basal cochlear turns from middle-aged GC-A WT mice and GC-A KO mice, with or without AT (Figure 6D).

Looking on IHC and auditory fibers, GC-A KO mice exhibit IHC synaptopathy and auditory neuropathy that is most pronounced for higher-frequency regions and that progresses over age and with AT.

### GC-A Mediated Poly (ADP-Ribose) Polymerase (PARP) Activity in the Organ of Corti and SG

**FIGURE 7:**
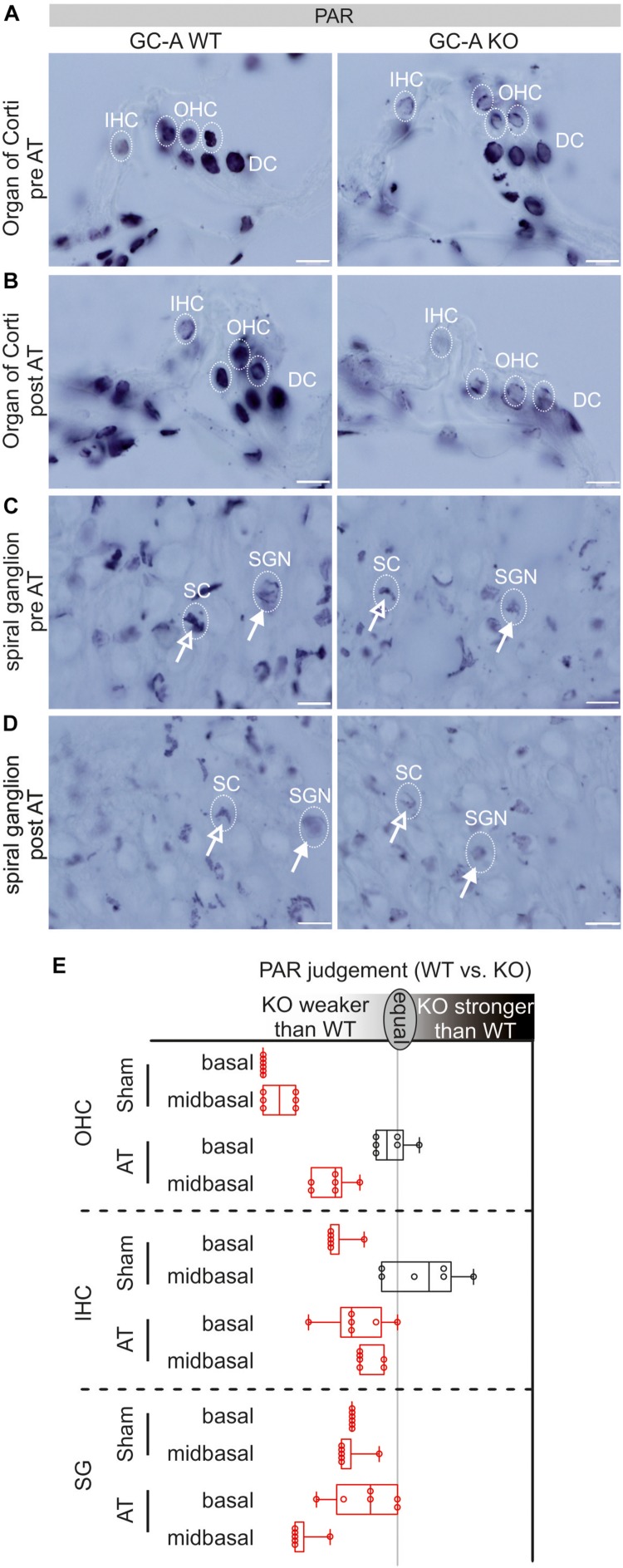
PAR-staining in organ of Corti and SG in GC-A WT and KO mice pre and post acoustic trauma. **(A)** Reduced PAR-staining of OHC nuclei but not DCs in the GC-A KO mouse. No difference was found for IHC in mid-basal turns. **(B)** After acoustic trauma, a reduction of PAR (not significant) was shown for IHC and OHCs in the GC-A KO mice, compared to WT. **(C)** PAR-staining in SG of SGNs (closed arrow) and satellite cells (SCs, open arrow) in sham-exposed GC-A WT and KO mice. **(D)** After acoustic trauma, GC-A KO animals had weaker PAR-staining of SCs and SGNs than WT mice. Scale bars: 10 μm. **(E)** Quantification of PAR-staining in OHC, IHC, and SG as evaluated by six independent referees; red colors indicate a reduction of PAR in GC-A. Boxplot shows median ± quartiles; whiskers mark the whole range.

To link the damaging effects of GC-A gene disruption on hair cell function with potential downstream effectors of the cGKI pathway, we studied the presence of PAR polymers, which were previously shown to be activated by elevated cGMP in cochlear hair cells (Jaumann et al., 2012). Cochleae of young GC-A WT and KO animals were analyzed before and after AT for possible differences in intracellular accumulation of PAR, with six ears from three animals judged by six persons in blind evaluations, and PAR was found to be either elevated or reduced in GC-A KO mice relative to GC-A WT mice (Figure 7E). In GC-A WT mice, a ubiquitous basal level of PAR was observed in nuclei of OHCs and IHCs, supporting the presence of Deiters’ cells (DCs) (Figure 7A, left panel) and SGN or satellite cells (SCs), respectively (Figure 7C, left panel). In OHCs, sham GC-A KO mice exhibited a decline in PARP activity in mid-basal and basal cochlear turns compared with GC-A WT mice (Figures 7A,E, left panel) but not in apical turns (not shown). After AT, the PAR accumulation in OHCs of GC-A KO mice was only reduced in the mid-basal turn compared to that in GC-A WT mice (Figures 7B,E, right panel). Reduced PARP activity, reduced DPOAE I/O, and reduced KCNQ4 surface expression were observed in young GC-A KO mice and may thus be regarded as an endogenous convergent downstream target of the GC-A-induced cGMP signaling pathway in OHCs. In IHCs or SGN/SCs from GC-A KO mice, a decline in PAR staining intensity was observed in mid-frequency cochlear turns as shown for IHCs in mid-basal turns (Figures 7A,E, left panel) or SGN/SCs in basal and mid-basal turns (Figures 7C,E, left panel).

However, in contrast to PAR in OHCs, at the IHC/SGN level, PAR staining decreased in GC-A KO mice compared with WT mice after AT, as shown for IHCs in basal and mid-basal cochlear turns (Figures 7B,E, right panel) and SGNs/SCs in mid-basal turns after trauma (Figures 7D,E).

The experiment could confirm that GC-A KO mice exhibit a differential reduction in PAR staining, likely caused by a decrease in PARP activity in OHCs and at the IHC, SGN, and SC level. The PARP-1 decline at the IHC/SGN level in GC-A KO mice may be part of the observed functional changes in GC-A KO mice at the auditory-nerve and IHC ribbon-synapse level.

**FIGURE 8:**
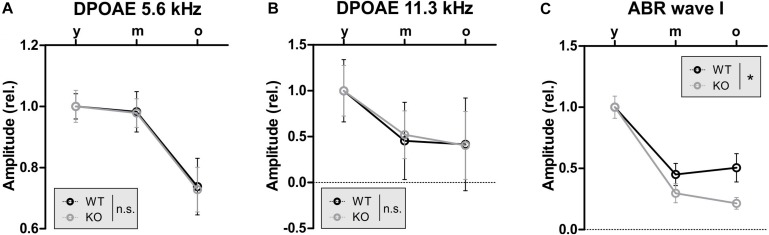
GC-A KO exhibit the same aging progress in OHC function as GC-A WT but accelerated reduction of auditory nerve responses over age. **(A,B)** DPOAE signal growth function in response to pure-tone sounds at f1 = 5.6 and 11.3 kHz were similarly reduced over age in GC-A KO and WT mice [5 kHz: two-way ANOVA, *F*(1,106) = 0.01, *P* = 0.928, *n* = 8–14/16–28 mice/ears each, 11 kHz: two-way ANOVA, *F*(1,106) = 0.00, *P* = 0.951, *n* = 8–14/16–28 mice/ears each]. **(C)** Noise-burst-evoked ABR wave I amplitude growth functions were more strongly reduced over age in GC-A KO than in WT mice [two-way ANOVA, *F*(1,71) = 4.72, *P* = 0.0033, *n* = 5–8/10–16 mice/ears each]. Mean ± SEM. ^∗^*P* < 0.05.

Thus far, the overall conclusion relies on GC-A-induced protective activities at the OHC level being independent of aging (and AT), while the GC-A-induced protective activities at the IHC/SGN level, reflected in ABR wave I changes, show evidence of being reinforced with age (or AT). To validate this idea, we analyzed the progression of age-related hearing loss in GC-A WT and KO mice for OHC function measured as DPOAE (Figures 8A,B and Table 2F) or IHC function measured as ABR wave I (Figure 8C). While GC-A KO mice exhibit the same aging process regarding OHC function measured with DPOAE I/O function in response to pure-tone sounds at f1 = 5.6 and 11.3 kHz (Figures 8A,B), the reduction in auditory-nerve responses was greater in GC-A KO mice as age increased (Figure 8C).

**FIGURE 9:**
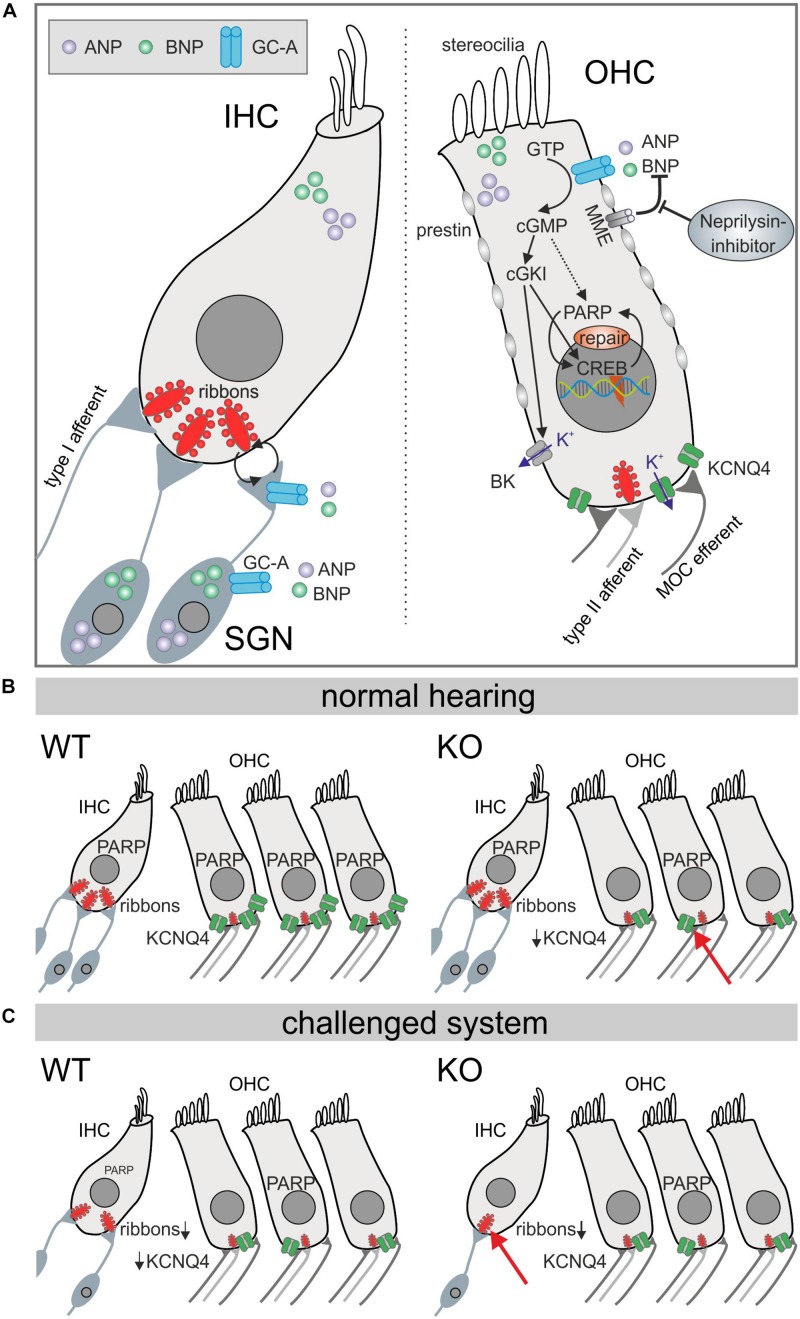
Diagram illustrating GC-A/cGMP signaling mechanisms in auditory hair cells. **(A)** Summery of GC-A dependent intercellular signaling in IHC, OHC, and SGNs. The natriuretic peptides ANP (violet) and BNP (bright green) both bind to the membrane bound GC-A (blue) in OHCs or the SGN and activate a cGMP dependent cascade that ends in PARP increase. The effects in IHCs are due to pre- and postsynaptic integrity. **(B)** In the basic hearing situation, the number of IHC ribbons is not reduced in GC-A KOs, but in OHCs, KCNQ4 is impaired which leads to a functional phenotype measureable in DPOAE growth functions. **(C)** However, in the challenged system after acoustic overexposure or in aged animals, the number of IHC ribbons is more reduced in GC-A KO mice compared with WT which is correlated with a decreased ABR wave I amplitude, while OHCs are unaffected.

In summary, these findings point to hair-cell-specific GC-A expression and function acting differentially in OHCs and IHCs during aging. OHC electromechanical properties in high-frequency cochlear turns are already diminished at a young age in the absence of GC-A, when KCNQ4 surface expression or PARP-1 levels are also reduced. Thereby, ANP and BNP, both expressed in OHCs, can act on GC-A in OHCs in an autocrine or paracrine manner. The data demonstrate that GC-A possibly maintains basic OHC function through cGMP/cGKI/cyclic AMP response-element binding (CREB), or PARP signaling independent of aging or AT (Figure 9). In contrast, at the IHC level, paracrine activation of GC-A signaling, possibly also through cGMP/cGKI/PARP, in SG feed-back to IHC nerve terminals and summed auditory nerve (ABR wave I) responses protects against noise/age-dependent hearing loss (Figure 9, IHC and SGN).

## Discussion

In the present study, we identified the particulate GC-A receptor (also named NPR-A) as an upstream regulator of otoprotective cGMP activities. We recognized hair-cell-specific differences in GC-A function in IHCs and OHCs with respect to normal hearing, aging, and AT-induced injury. In line with our hypothesis, we present clear evidence that GC-A receptor ligands have a crucial function for maintaining OHC’s and IHC’s pre-postsynaptic integrity, particularly in high-frequency cochlear turns. The therapeutic value of these findings is significant, since neprilysin-inhibitors (the peptidase responsible for degrading ANP and BNP, which are ligands for GC-A) are safe and well-tolerated drugs already used for chronic therapy in heart-failure patients (Feygina et al., 2019).

### Expression of GC-A and Its Ligands in the Cochlea

Using isolated hair cells and SG of the mature cochlea, we identified the NPs ANP and BNP as well as GC-A in OHCs and SG and confirmed the presence of NPs in IHCs, corroborating previous studies (Meyer zum Gottesberge et al., 1991; Yoon and Anniko, 1994; Suzuki et al., 1998, 2000; Kuhn, 2003, 2009; Schulz, 2005; Kemp-Harper and Feil, 2008; Kleppisch and Feil, 2009; Alexander et al., 2011; Qiao et al., 2011; Sun et al., 2013, 2014; Shen et al., 2015; Möhrle et al., 2017; Fitzakerley and Trachte, 2018). We could not detect GC-A in IHCs. This suggests a possible autocrine or paracrine NP/GC-A effect on OHCs and SG, whereas GC-A affects IHC synapses, likely indirectly through retrograde signaling from SGNs on IHCs. Retrograde signaling between the IHC presynapse and auditory nerve postsynapse (Kujawa and Liberman, 2009) is suggested from AT-induced damage of IHC pre- and postsynapses, possibly including signaling cascades from SCs (Sugawara et al., 2005; Wan et al., 2014).

### GC-A KO Mice Exhibit OHC Impairment Independent of Acoustic Trauma and Aging

In the present study, GC-A KO mice were shown to exhibit a normal hearing threshold, reflected through normal thresholds of DPOAEs. However, already at young ages, the shallow growth of the DPOAE I/O function (Figure 3) indicated a loss of OHC electromotility in response to higher frequency (11.3 kHz) stimuli. This phenotype of GC-A KO mice was not aggravated after AT (Figure 4) or with aging (Figure 8). This suggests that GC-A in OHCs exhibits endogenous, basal otoprotective activity. If this is lost, OHCs lose their proper functional phenotype. GC-A/cGMP signaling may maintain the functional OHC phenotype through different downstream cascades:

(i) Already at a young age, GC-A KO mice had developed diminished electromechanical properties of OHCs; not, however, at threshold, but at higher sound levels in high-frequency cochlear regions, where a stronger K^+^ influx through the stereocilial MET-channels needs to be managed. This was associated with a visible loss of KCNQ4 type K^+^ channels on the surface OHC membranes. KCNQ4 channels mediate the major OHC K^+^ current *I*_K,n_ at rest and thus determine the membrane potential and time constant (Housley and Ashmore, 1992; Marcotti and Kros, 1999; Kharkovets et al., 2000, 2006). When KCNQ4 is not functional in OHCs, e.g., in non-syndromic autosomal dominant (DFNA2) patients or mouse models with mutation of KCNQ4 (Jentsch, 2000; Kharkovets et al., 2000; Gao et al., 2013), progressive high-frequency hearing loss linked to OHC loss develops. Furthermore, dysfunction of KCNQ4 contributes to noise- and age-dependent high-frequency hearing loss (Van Eyken et al., 2006). Questioning how GC-A may influence KCNQ4 surface expression, the obvious need for fast repolarization of OHCs following intense and high-frequency stimulation, to keep KCNQ4 proteins in place, should be considered. The function of the big potassium (BK) channel is known to be associated with maintenance of KCNQ4 channel expression (Rüttiger et al., 2004). BK is typically activated through efferent inhibition of OHCs that works via the unusual combination of Ca^2+^ influx through the acetylcholine receptor AChRa9/10 (Weisstaub et al., 2002). AChRa9/10 mediates Ca^2+^ influx that leads to BK activation, which triggers K^+^ conductance (Oliver et al., 2000; Maison et al., 2013). Indeed, large-conductance BK channels can be activated through cGMP/cGKI-induced phosphorylation (Zhou et al., 2010; Kyle et al., 2013), providing a mechanism by which endogenous GC-A/cGMP activities might contribute to maintaining stable OHC function in high-frequency regions under high sound intensities (Figure 9) (Rüttiger et al., 2004; Beisel et al., 2005; Engel et al., 2006). As posttraumatic loss of KCNQ4 in OHCs was prevented by elevation of cGMP levels through PDE5 inhibition with vardenafil (Jaumann et al., 2012), cGMP was predicted to rescue OHCs by maintaining OHC membrane potential and membrane time constants in high-frequency regions during exposure to traumatic sound intensities (Jaumann et al., 2012). However, here, we observed a GC-A effect independent of AT and age in OHCs, suggesting that a GC-A-independent cGMP generator pathway, in addition to GC-A/cGMP/cGKI signaling, may contribute to aging and AT vulnerability in OHCs.

(ii) Alternatively, GC-A/cGMP/cGKI-induced signaling may positively influence OHC stability through phosphorylation of the transcription factor CREB as previously described (Fiscus, 2002). A well-known downstream target of CREB is PARP-1, a polymerase mediating PolyADP-ribosylation. PAR polymers are products of PARP activity, which has been shown to be involved in DNA repair and transcriptional activity in a cGMP- and cGKI-dependent manner, independent of CREB (Kim et al., 1999; Paquet-Durand et al., 2007). PARP was also shown to be directly activated by cGMP (Paquet-Durand et al., 2007; Sahaboglu et al., 2010). CREB and cGMP-induced activation of PARP is suggested to exhibit survival and anti-aging potential (Beneke and Burkle, 2007). During this process, enhanced cell stability or survival induced by activated PARP was suggested to be based on the counteracting of ongoing cellular DNA breaks by PARP, which facilitates transcription, replication, and DNA base-excision repair (Yu et al., 2006).

In conclusion, a reduction in KCNQ4 and PARP in OHCs was observed in young GC-A KO mice in comparison to GC-A WT mice. Both KCNQ4, via BK activation, and PARP-1 activity may be part of endogenous GC-A/cGMP-induced protective signaling cascades that help maintain the basal OHC phenotype and function following AT during metabolically demanding conditions.

### GC-A KO Mice Exhibit Enhanced IHC Synaptopathy and Auditory Neuropathy in Response to Acoustic Trauma and Aging

In contrast to OHCs, where the negative effect of GC-A gene disruption is not reinforced by AT or aging, the impact of GC-A inactivation on IHC synapses and SGN integrity was more pronounced following AT and over the lifespan. The absence of GC-A expression in isolated IHCs suggests that the observed age- and AT-induced reductions in ribbon numbers in IHC synapses in GC-A KO mice occur secondarily through damage of SGNs. Postsynaptic excitotoxicity events are suggested to lead to deafferentation during age- and NIHL (Pujol and Puel, 1999; Kujawa and Liberman, 2009). SC signaling (Sugawara et al., 2005) may secondarily affect IHC synapses in a similar manner, as we predicted here for IHC synapse damage in GC-A KO mice. Although we cannot exclude subthreshold expression of GC-A that remained undetected in IHCs, the present study argues on the assumption that IHC synapse damage in GC-A KO mice is the result of a GC-A/cGMP/cGKI/PARP cascade in SG.

In GC-A KO mice, auditory neuropathy is reflected as a loss of IHC ribbons in higher-frequency cochlear turns. This loss is associated with a reduction in the summed response of the auditory nerve (ABR wave I amplitude), indicating an auditory neuropathy in GC-A KO mice that is most pronounced in middle-aged and old mice and after AT. In young GC-A KO mice, the number of IHC synaptic ribbons in high-frequency cochlear regions was already reduced. This decline was not yet translated to reduced auditory-nerve responses, but was already accompanied by reduced PAR in the SG in these regions (Figure 8). If the observed worsening and acceleration of IHC synapse damage and loss of ABR wave I amplitude after AT and during aging in GC-A KO mice is reflected in altered PAR accumulation, this would need further inspection.

In conclusion, this finding reveals a clear role for GC-A ligands in maintaining basic IHC synapse function and pre- and postsynaptic integrity of IHC in high-frequency cochlear regions during aging and AT. The metabolic sensitivity of IHC synapses and their contribution to hidden and age-dependent hearing loss is thereby confirmed (Keithley, 2019; Lee et al., 2019). This also confirms our initial hypothesis that GC-A ligands act as possible key regulators of energy consumption and metabolism to maintain hearing function.

### Considerations of the Therapeutic Value of GC-A Signaling

Based on these results, future studies should focus on the potential of enhancing ANP/GC-A/cGMP signaling for restoration of normal hearing to counteract hidden hearing loss and IHC synaptopathy, as well as age-related hearing loss or NIHL. Here, efforts to stimulate GC-A through the ligand ANP may be particularly promising because (i) ANP levels in endolymph are two orders of magnitude higher than in plasma (Yoon et al., 2012); (ii) the ANP-producing serine protease corin is expressed in the cochlea, indicating that cochlear cells are capable of converting proANP to ANP (Labine et al., 2015; Fitzakerley and Trachte, 2018); and (iii) preliminary studies pointed to a transient improvement in hearing thresholds following systemic ANP administration (Yoon et al., 2015).

Alternatively, stimulation with the GC-A ligand BNP may be considered. While BNP was not found to be expressed in the cochlea (Fitzakerley and Trachte, 2018), the present study clearly indicates BNP expression in hair cells and SG of the adult murine cochlea. Interestingly, in this context, BNP has been shown to increase the open probability of BK channels and to suppress the membrane excitability of small-sized dorsal-root-ganglion neurons (Li et al., 2016). As BK is stimulated through cGKI signaling (Zhou et al., 1998; Frankenreiter et al., 2017) and BK channel activation is predicted to possibly counteract excitotoxic events in hair cells (Rüttiger et al., 2004; Engel et al., 2006, see above), a BNP/GC-A/cGMP/cGKI/BK cascade may also contribute to the observed GC-A otoprotective functions.

Moreover, because the cGMP-degrading enzyme PDE9a might be a target for drugs that increase cGMP pools that are predominantly controlled by ANP/GC-A (Lee et al., 2015), we searched for and found PDE9a expression in the cochlea. Therefore, PDE9a inhibitors should be included as potential pharmaceutical drug candidates for the inner ear in future studies.

Finally, inhibition of the NP-degrading enzymes, e.g., membrane metalloendopeptidase (MME) (also called neprilysin or neutral endopeptidase), that typically reduce cGMP production through GC-A should be considered. Indeed, MME mRNA was found to be expressed in hair cells and possibly in SG (Shen et al., 2015; Fitzakerley and Trachte, 2018), but the protective potential of MME inhibition against AT or age-dependent hearing loss has not yet been tested.

### Hypertension in GC-A KO Mice

GC-A KO mice develop arterial hypertension (Kuhn, 2005). Whether arterial hypertension itself may contribute to age-related hearing loss is still controversial (Przewozny et al., 2015; Soares et al., 2016; Reed et al., 2019). Although we cannot exclude the possibility that glucose metabolism may be altered in the GC-A KO mice, and consequently affect hearing, other mouse mutants with hypertension (NO-GC KO mice) have normal and persisting hearing function (Friebe et al., 2007; Möhrle et al., 2017). However, it is advisable to consider whether proper blood flow and glucose metabolism participates in the age- and AT-related pre- and postsynaptic deficits observed here in GC-A KO mice. A normal metabolic supply is required for sustained and untiring vesicle release, particularly in high-frequency cochlear regions. The use of tissue-specific KO mouse mutants may help to avoid the hypertensive phenotype in future studies.

## Conclusion

The present longitudinal study of GC-A KO mice strongly supports our initial hypothesis that GC-A signaling may contribute to the metabolic supply of OHCs. Thus, we could demonstrate that GC-A maintains endogenous OHC stability, and contributes to AT- and age vulnerability of IHC and auditory-nerve function. The protective GC-A effect on hearing thereby differs profoundly from that of GC-B and NO-GC. The deletion of GC-B leads to diminished temporal auditory processing likely through affecting efferent feedback loops (Wolter et al., 2018). In contrast, the loss of NO-GC may have positive effects: The deletion of NO-GC subtype 1 and 2 leads to slight protection of OHCs, IHCs, and auditory nerve function after noise damage (Möhrle et al., 2017). In conclusion, the observed otoprotective functions of elevated cGMP levels previously achieved through PDE 5 inhibition (Jaumann et al., 2012) may have a major GC-A contribution. Augmenting NP/GC-A signaling should be considered as a protective therapy for hearing preservation.

## Data Availability Statement

The raw data supporting the conclusions of this article will be made available by the authors, without undue reservation, to any qualified researcher.

## Ethics Statement

Animal care, procedures, and treatments were performed in accordance with institutional and national guidelines following approval by the University of Tübingen, Veterinary Care Unit, and the Animal Care and Ethics Committee of the regional board of the Federal State Government of Baden-Württemberg, Germany, and followed the guidelines of the EU Directive 2010/63/EU for animal experiments.

## Author Contributions

PM, DM, MaK, and LR contributed to conceptualization and writing. PM, DM, and LR contributed to analysis. PM, DM, PE, KR, SW, AT, IL, and MW contributed to investigation. MaK and LR contributed to supervision. RF, JE, FP-D, MiK, LR, and MaK contributed to review and editing.

## Conflict of Interest

The authors declare that the research was conducted in the absence of any commercial or financial relationships that could be construed as a potential conflict of interest.

## Footnotes

1 https://www.nia.nih.gov/health/hearing-loss-common-problem-older-adults

## Supplementary Material

FIGURE S1 Slopes of growth functions of DPOAE signals and regressions on DPOAEs loss in GC-A WT and GC-A KO mice. **(A)** Shifts of DPOAE signal growth functions in response to pure-tone sounds at f1 = 5.6 kHz (left panel) were similar between GC-A KO and WT mice [two-way ANOVA, *F*(1,121) = 0.03, *P* = 0.8693, WT *n* = 3/6 mice/ears KO *n* = 4/7 mice/ears], while GC-A KO mice had smaller shifts after acoustic trauma for pure-tone sounds with f1 = 11.3 kHz [middle panel, two-way ANOVA, *F*(1,180) = 6.06, *P* = 0.0148, WT *n* = 3/6 mice/ears KO *n* = 4/8 mice/ears]. **(B)** To normalize the DPOAE I/O shift for respective frequencies, and to account for genotype differences preceding acoustic trauma induction, the regression between the measured DPOAE signal (in dB SPL) after acoustic trauma and the loss of DPOAE signal (in dB SPL) was calculated. The regression lines were not different between GC-A WT and KO mice with f1 = 5.6 kHz [left panel: unpaired two-tailed student’s *t*-test *t*(183) = 0.226, *P* = 0.98, WT *n* = 85 KO *n* = 102] and 11.3 kHz [middle panel: unpaired two-tailed student’s *t*-test *t*(69) = 0.027, *P* = 0.98, WT *n* = 28 KO *n* = 45], indicating similar relative loss of slope of the DPOAE I/O function. Mean ± SEM.
