# Supplementary figures and images for: Guanylyl Cyclase A/cGMP Signaling Slows Hidden, Age- and Acoustic Trauma-Induced Hearing Loss
Source: Front Aging Neurosci. 2020 Apr 9;12:83. doi: 10.3389/fnagi.2020.00083 (PMC7160671; doi:10.3389/fnagi.2020.00083)

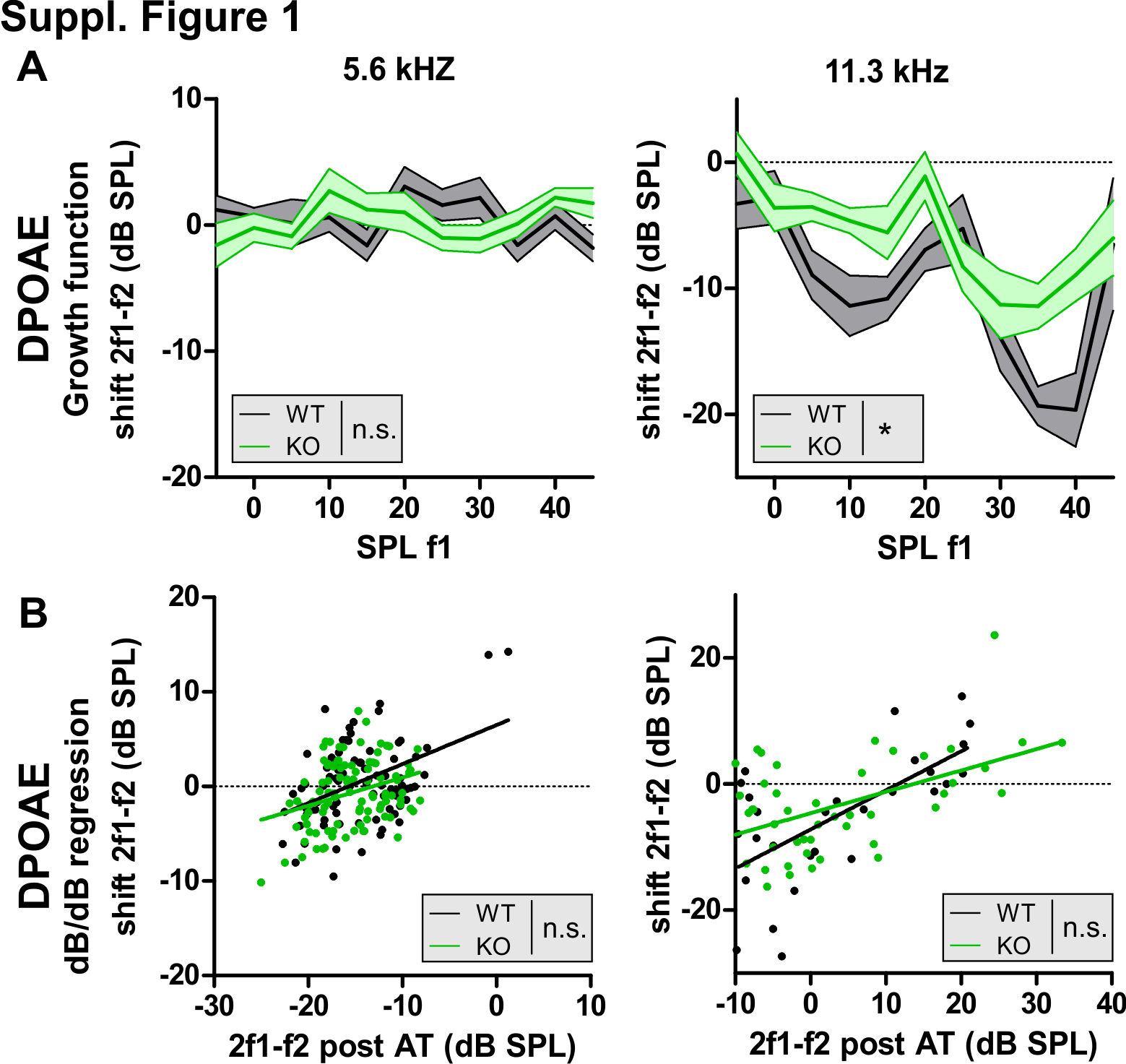

Supplement: FIGURE S1 — Slopes of growth functions of DPOAE signals and regressions on DPOAEs loss in GC-A WT and GC-A KO mice. (A) Shifts of DPOAE signal growth functions in response to pure-tone sounds at f1 = 5.6 kHz (left panel) were similar between GC-A KO and WT mice [two-way ANOVA, F(1,121) = 0.03, P = 0.8693, WT n = 3/6 mice/ears KO n = 4/7 mice/ears], while GC-A KO mice had smaller shifts after acoustic trauma for pure-tone sounds with f1 = 11.3 kHz [middle panel, two-way ANOVA, F(1,180) = 6.06, P = 0.0148, WT n = 3/6 mice/ears KO n = 4/8 mice/ears]. (B) To normalize the DPOAE I/O shift for respective frequencies, and to account for genotype differences preceding acoustic trauma induction, the regression between the measured DPOAE signal (in dB SPL) after acoustic trauma and the loss of DPOAE signal (in dB SPL) was calculated. The regression lines were not different between GC-A WT and KO mice with f1 = 5.6 kHz [left panel: unpaired two-tailed student’s t-test t(183) = 0.226, P = 0.98, WT n = 85 KO n = 102] and 11.3 kHz [middle panel: unpaired two-tailed student’s t-test t(69) = 0.027, P = 0.98, WT n = 28 KO n = 45], indicating similar relative loss of slope of the DPOAE I/O function. Mean ± SEM. [file Image_1.TIF]
